# An evolutionary portrait of the progenitor SARS-CoV-2 and its dominant offshoots in COVID-19 pandemic

**DOI:** 10.1093/molbev/msab118

**Published:** 2021-05-04

**Authors:** Sudhir Kumar, Qiqing Tao, Steven Weaver, Maxwell Sanderford, Marcos A Caraballo-Ortiz, Sudip Sharma, Sergei L K Pond, Sayaka Miura

**Affiliations:** 1 Institute for Genomics and Evolutionary Medicine, Temple University, Philadelphia, PA; 2 Department of Biology, Temple University, Philadelphia, PA; 3 Center for Excellence in Genome Medicine and Research, King Abdulaziz University, Jeddah, Saudi Arabia

## Abstract

Global sequencing of hundreds of thousands of genomes of Severe acute respiratory syndrome coronavirus 2, SARS-CoV-2, has continued to reveal new genetic variants that are the key to unraveling its early evolutionary history and tracking its global spread over time. Here, we present the heretofore cryptic mutational history and spatiotemporal dynamics of SARS-CoV-2 from an analysis of thousands of high-quality genomes. We report the likely most recent common ancestor of SARS-CoV-2, reconstructed through a novel application and advancement of computational methods initially developed to infer the mutational history of tumor cells in a patient. This progenitor genome differs from genomes of the first coronaviruses sampled in China by three variants, implying that none of the earliest patients represent the index case or gave rise to all the human infections. However, multiple coronavirus infections in China and the USA harbored the progenitor genetic fingerprint in January 2020 and later, suggesting that the progenitor was spreading worldwide months before and after the first reported cases of COVID-19 in China. Mutations of the progenitor and its offshoots have produced many dominant coronavirus strains, which have spread episodically over time. Fingerprinting based on common mutations reveals that the same coronavirus lineage has dominated North America for most of the pandemic in 2020. There have been multiple replacements of predominant coronavirus strains in Europe and Asia and the continued presence of multiple high-frequency strains in Asia and North America. We have developed a continually updating dashboard of global evolution and spatiotemporal trends of SARS-CoV-2 spread (http://sars2evo.datamonkey.org/).

## Introduction

The early evolutionary history of SARS-CoV-2, which causes COVID-19, remains unclear despite an unprecedented scope of global genome sequencing of Severe Acute Respiratory Syndrome Coronavirus 2 (SARS-CoV-2) and a multitude of phylogenetic analyses (Forster et al. 2020; Lemey et al. 2020; Rambaut, Holmes, et al. 2020; Tang et al. 2020; Worobey et al. 2020; Chiara et al. 2021; Komissarov et al. 2021; Lemieux et al. 2021; da Silva Filipe et al. 2021) (Pekar et al. 2021). Sophisticated investigations have shown that traditional molecular phylogenetic analyses do not produce reliable evolutionary inferences about the early history of SARS-CoV-2 due to low sequence divergence, a limited number of phylogenetically informative sites, and the ubiquity of sequencing errors (De Maio et al. 2020; Mavian et al. 2020; Turakhia et al. 2020). In particular, the root of the SARS-CoV-2 phylogeny remains elusive (Morel et al. 2020; Pipes et al. 2020) because the closely-related non-human coronavirus (outgroups) are more than 1,100 base differences from human SARS-CoV-2 genomes, as compared to fewer than 30 differences between human SARS-CoV-2 genomes' sequenced early on (December 2019 and January 2020) (Andersen et al. 2020; Castells et al. 2020; Gómez-Carballa et al. 2020; Lai et al. 2020; Mavian et al. 2020; Morel et al. 2020; Pipes et al. 2020; Wenzel 2020). Without a reliable root of the SARS-CoV-2 phylogeny, the most recent ancestor sequence cannot be accurately reconstructed, and it is not possible to assess the genetic diversity of SARS-CoV-2 that existed at the time of its first outbreak. Consequently, we cannot determine if any of the coronaviruses isolated to date carry the genome of the progenitor of all human SARS-CoV-2 infections. Knowing the progenitor genome will also help determine how close the earliest patients sampled in China are to "patient zero," i.e., the first human transmission case.

The orientation and order of early mutations giving rise to common coronavirus variants will also be compromised if the earliest coronavirus isolates are incorrectly used to root the SARS-CoV-2 phylogenies (Dearlove et al. 2020; Fauver et al. 2020; Stefanelli et al. 2020; Tang et al. 2020). Some investigations of COVID-19 patients and their coronaviruses' genomes already reported the presence of multiple variants (Lu et al. 2020), and genomes of viral samples from December 2019 in China had as many as five differences. These observations require an explicit test of the assumption that one of the early sampled coronavirus genomes was the progenitor of all the strains infecting humans.

Traditionally, ancestral sequences are estimated using a rooted phylogeny (Yang et al. 1995; Nei and Kumar 2002). This ancestral sequence can then be compared with the sequenced genomes to locate the one that is most similar to the inferred progenitor and/or placed closest to the root in the phylogeny. However, as noted above, attempts using *ad hoc* and traditional methods are fraught with difficulties and have not produced consistent and robust results (Morel et al. 2020; Pipes et al. 2020). Some methods also incorporate sampling times in phylogenetic inference, but they will automatically favor placing the earliest sampled genomes at or near the root of the tree, which introduces circularity in testing the hypothesis that the earliest sampled genomes were ancestral because sampling time is used in the inference procedure (Pipes et al. 2020; Pekar et al. 2021).

## Results and Discussion

### A mutational order approach for SARS-CoV-2

We applied a mutation order approach (MOA) to directly reconstruct the ancestral sequence and the mutational history of genomes (Jahn et al. 2016; Ross and Markowetz 2016; Miura et al. 2018). MOA does not infer phylogeny as an intermediate step and is often used to build the evolutionary history of tumor cells that evolve clonally and without recombination. This approach is suitable for analyzing SARS-CoV-2 genomes because of their quasi-species evolutionary behavior (clonal) and the lack of evidence of significant recombination within human outbreaks (Richard et al. 2020), both of which preserve the collinearity of variants in genomes. While reports of recombination among circulating SARS-CoV-2 genomes have begun to appear (Jackson et al. 2020), the fraction of circulating recombinant strains is likely very small and geographically limited and will not affect analyses conducted on sequences sampled in 2020. Recombination also poses difficulties to phylogenetic methods since a single tree is no longer suitable for describing evolutionary relationships. This feature permits effective use of shared co-occurrence of variants in genomes and the frequencies of individual variants for inferring the mutational history, notwithstanding the presence of sequencing errors and other artifacts (Kim and Simon 2014; Jahn et al. 2016) (see *Materials and Methods*).

We advanced MOA to make it applicable for analyzing SARS-CoV-2 genomes. First, the normal cell sequence in tumors provides the ancestral (non-cancerous) genome sequence to orient the mutational changes, but such a direct ancestor is not available for coronaviruses in which the closest outgroup sequences are over 30-times more different than any two SARS-CoV-2 strains. Second, SARS-CoV-2 genomes may not satisfy the perfect phylogeny assumption of MOA analysis because some genomic positions have likely experienced multiple and recurrent mutations (van Dorp et al. 2020; Martin et al. 2021). So, we used shared co-occurrence of variants among genomes to assess the impact of the violation of the perfect phylogeny assumption and select mutation orientations and histories in the maximum likelihood approach. We also devised a bootstrap procedure to place confidence limits on the inferred mutation order in which bootstrap replicate datasets are generated by sampling genomes with replacement.

Here we present results from analyses of two snapshots of the fast-growing collection of SARS-CoV-2 genomes to make inferences and assess the robustness of the inferred mutational histories to the growing genome collection, expanding at an unprecedented rate. We first present results from the 29KG dataset and then evaluate the concordance of the mutational history inferred by using an expanded 68KG dataset, which establishes that the conclusions are robust to the sampling of genomes. The first snapshot was retrieved from GISAID (Shu and Mccauley 2017) on July 7, 2020, and consisted of 60,332 genomes. Of these, 29,681 were selected because they were longer than the 28,000 bases threshold imposed (29KG dataset) and did not include an excessive number of unresolved bases in any genomic regions. This second snapshot was acquired on October 12, 2020, from GISAID and contained 133,741 genomes, of which 68,057 genomes met the inclusion criteria (68KG dataset).

We then applied mutational fingerprints inferred using the 68KG dataset to an expanded dataset of 172,480 genomes (sampled on December 30, 2020; 172KG) to track global spatiotemporal dynamics SARS-CoV-2. We have also set up a live dashboard showing regularly updated results because the processes of data analysis, manuscript preparation, and peer-review of scientific articles are much slower than the pace of expansion of SARS-CoV-2 genome collection. Also, we provide a simple "in-the-browser" tool to classify any SARS-CoV-2 genome based on key mutations derived by the MOA analysis (http://sars2evo.datamonkey.org/).

## Mutational history and progenitor of SARS-COV-2

We used MOA to reconstruct the history of mutations that gave rise to 49 common single nucleotide variants (SNVs) in the 29KG dataset. These variants occur with greater than 1% variant frequency (*vf* > 1%) – a threshold chosen to avoid including variants that may be due to sequencing errors (Fig. 1a). To simplify notation, we used the inferred mutation history to denote key groups of mutations by assigning Greek symbols (μ, ν, α, β, γ, δ, and ε) to them. Individual mutations were assigned numbers and letters based on the reconstructed order and their parent-offspring relationships (Table 1). We estimated the timing of mutation for each mutation based on the timestamp of the viral samples' genome sequences in which it first appeared (Table 1, see *Materials and Methods*). The inferred mutation order was in excellent agreement with the temporal pattern of the first appearance of variants in the 29KG dataset. The timestamp of 47 out of 49 mutations was greater than or equal to the timestamp of the corresponding preceding mutation in mutational history. The exceptions were seen for two low-frequency offshoot mutations ($\beta_{3b}$ and $\beta_{3c}$; see *Materials and Methods*). This concordance provides independent validation of the reconstructed mutation graph because neither sampling dates nor locations were used in MOA analysis.

In the mutational history, new variants occurred in the genomic background of the variants preceding them in the reconstructed mutation history with a very high propensity (co-occurrence index, COI > 84%; Fig. 2), except for one low-frequency offshoot mentioned above ($\beta_{3b}$; COI = 54%). Overall, these results suggest a strong signal to infer a sequential mutational history, even though a small minority of sequences at a position may have experienced homoplasy or recurrent mutations. Indeed, a bootstrap analysis involving genome resampling to assess the robustness of the mutation history produced high bootstrap confidence levels (BCLs) for key groups of mutations as well as many offshoots (Fig. 2; BCL > 95%).

### *Episodic evolution and selection*

The order of some mutations in the mutational history is not established with high BCLs, e.g., the relative order of $\varepsilon_1$, $\varepsilon_2$, and $\varepsilon_3$ mutations. This is because the three ε variants almost always occur together (7,624 genomes), and the intermediate combinations of ε variants were found in only 42

genomes. Similarly, the count of genomes harboring all three β variants (22,739 genomes) far exceeded those with two or fewer β variants (201 genomes). There is a strong temporal tendency of variants to be sampled together (e.g., $\varepsilon_1$ - $\varepsilon_3$ and $\alpha_{1a}$ - $\alpha_{1d}$), suggesting an episodic spread of variants (Wald–Wolfowitz runs-tests $P$ << 0.01; see *Materials and Methods*) that does not allow for determining the precise order of mutation appearance. Episodic variant spread may be caused by founder effects, positive selection, or both (e.g., MacLean et al. 2020). It may also be an artifact of highly uneven regional and temporal genome sequencing that will produce a biased representative sample of the actual worldwide population (Fig. 1b).

In this mutation history, the ratio of nonsynonymous to synonymous changes (N/S) is 1.9, which is almost 10-times larger than the ratio of 0.18 for the inferred proCoV2 and observed Bat CoV proteins. The McDonald-Kreitman test (McDonald and Kreitman 1991) rejected the similarity of molecular evolutionary patterns observed within the SARS-CoV-2 population (29KG dataset) and between human proCoV2 and the bat coronavirus. However, the selective interpretation of such a difference is complicated by the fact that polymorphisms in SARS-CoV-2 genomes are affected by molecular mechanisms (e.g., RNA editing) (Giorgio et al. 2020; Rice et al. 2020), not just selection, and slightly deleterious alleles can become common when there is a population expansion (Casals and Bertranpetit 2012). Furthermore, selection may have played a significant role during the divergence of human CoV-2 and bat CoV sequences (MacLean et al. 2021; Martin et al. 2021; Tegally et al. 2021). Nevertheless, N/S patterns derived from common variants show that molecular evolutionary patterns observed within SARS-CoV-2 genomes infecting humans differ from those spanning the divergence between the bat RaTG13 and SARS-CoV-2 genomes, even though positive selection in the early SARS-CoV-2 pandemic history may have been limited (Chiara et al. 2021; MacLean et al. 2021).

*The progenitor genome and the index case*

The root of the mutation tree is the most recent common ancestor (MRCA) of all the genomes analyzed, which gave rise to two early coronavirus lineages (ν and α; Fig. 2). The MRCA genome was the progenitor of all SARS-CoV-2 infections globally, henceforth proCoV2, and was likely carried by the *first detectable* case of human transmission in the COVID-19 pandemic (index case). A comparison of proCoV2 with Wuhan-1 genomes revealed three differences in 49 positions analyzed, which was also true for other reference genomes (Fig. 1c). This suggests that the Wuhan-1 (EPI_ISL 402123) and the other earliest sampled genomes are derived coronavirus strains that arose from proCoV2 after the divergence of ν and α lineages (Fig. 2). According to the mutational history, the Wuhan-1 strain evolved by three successive α mutations (two synonymous and one nonsynonymous) in proCoV2 ($\alpha_1$, $\alpha_2$, and $\alpha_3$). This progression is statistically supported (BCL = 100%), which is made possible by the presence of 896 intermediate genomes containing one or two α variants in the 29KG dataset. Importantly, three closely related non-human coronavirus genomes (bats and pangolin) all have the same base at these positions as does the proCoV2 genome, suggesting that the ancestral genome did not contain α variants. Furthermore, genomes with ν variants of proCoV2 do not contain the other 47 variants, all of which occurred on the genomic background containing $\alpha_1$-$\alpha_3$. These facts support the inference that coronaviruses lacking α variants were the ancestors of Wuhan-1 and other genomes sampled in December 2019 in China (Fig. 1c). Therefore, we conclude that Wuhan-1 was not the direct ancestor of all the early coronavirus infections globally.

A comparison of the proCoV2 genetic fingerprint (49 positions) in the 29KG collection revealed three matches in China (Fujian, Guangdong, and Hangzhou) and three in the USA (Washington) in January 2020 (Fig. 1c). One more match was found in New York in March 2020. The ν mutant of proCoV2 was first sampled 59 days after the Wuhan-1 strain. This means that the progenitor coronavirus spread and mutated in the human population for months after the first reported COVID-19 cases. Furthermore, comparisons of the protein sequences encoded by the proCoV2 genome revealed 131 other genomic matches, which contained only synonymous differences from proCoV2. A majority (89 genomes) of these matches were from coronaviruses sampled in China and other Asian countries (Fig. 1d). The first sequence was sampled 12 days after Wuhan-1. Multiple matches were found in all sampled continents and detected as late as April 2020 in Europe. These spatiotemporal patterns suggest that proCoV2 already possessed the repertoire of protein sequences needed to infect, spread, and persist in the global human population (see also MacLean et al. 2020).

*Coronavirus diversity before the first coronavirus outbreak*

The progenitor of all genomes sequenced from human coronavirus infections (MRCA, proCoV2) is three bases different from the Wuhan-1 genome, which extends the ancestry before late November/early December 2020 date suggested by Pekar et al. (2021). Their inference was based on analyzing SARS-CoV-2 genomes from the first four months of coronavirus infections in China, with a strict molecular clock in which they placed coronavirus genomes from December 2019 are at the root of their phylogeny (Pekar et al. 2021). Their most likely root position is the same as the Wuhan-1 position in our mutation history (Fig. 1 and 3), which is not surprising because their dataset did not include more than 1000 genomes that comprise the early diverging ν lineage (Fig. 3). The genomes containing the ν lineage, sampled in North America, descended from an earlier ancestor that also gave rise to the genome (α lineage) at the root of Pekar et al. (2021) phylogeny. Therefore, our analysis has revealed an earlier MRCA than that detectable by considering a smaller subset of sequences from China.

The mutational history from proCoV2 to Wuhan-1 genome points to the presence of measurable coronavirus diversity before the earliest recognized coronavirus outbreak in December 2019 (Fig. 1). The presence of such diversity has been acknowledged and analyzed, e.g., Pekar et al. (2021), but variants present in this diverse population were not identified. Our analyses clearly show that the ancestors of the Wuhan-1 genome gave rise to a diversity of Wuhan-1's sibling coronavirus lineages ($\alpha_{3a}$ - $\alpha_{3j}$; Fig. 1 and 3). These sibling coronavirus lineages were detected in China in January 2020 ($\alpha_{3b}$ and $\alpha_{3f}$) and February 2020 in Asia ($\alpha_{3c}$ - $\alpha_{3e}$) and Europe ($\alpha_{3a}$, $\alpha_{3g}$ - $\alpha_{3j}$) (Table 1). Thousands of genomes in the 29KG dataset belong to siblings and ancestors of Wuhan-1 (Table 1, Fig. 3; yellow box). However, the paucity of genomes sampled in 2019 makes it impossible to establish the date and location of origin precisely, but some must have originated before the first detection of the outbreak. Notably, the evolution of $\alpha_3$ was preceded by the evolution of $\alpha_2$ and $\alpha_1$ lineages, with $\alpha_1$ spawning multiple offshoots first detected in Europe in February 2020 ($\alpha_{1a}$ - $\alpha_{1d}$). The ν lineage, detected in the USA in February 2020, is an even earlier descendant of proCoV2 and is a sibling of the α lineage (Table 1, Fig. 3). These lineages may not have been detected earlier because of the lack of sequencing in 2019, and it is likely that some originated early and spread around the world, whereas others evolved from proCoV2 or its early descendant in different parts of the world. Again, thousands of these coronavirus genomes were found throughout the world (Fig. 3; yellow box). None of these genomes contained the widely-studied Spike protein mutant (D614G), a β mutation that occurred in the genomes carrying all three α variants and was first seen in late January 2020. Therefore, the proCoV2 (MRCA) and a large diversity of its early descendants were all able to spread in the global human population.

*Estimated timing of MRCA (tMRCA) and the index case*

Because proCoV2 is three bases different from the Wuhan-1 genome, we estimate that the divergence of the earliest variants of proCoV2 occurred 5.8 - 8.1 weeks earlier, based on the range of estimated mutation rates of coronavirus genomes (see *Materials and Methods*). This timeline puts the presence of proCoV2 in late-October 2019, which is consistent with the report of a fragment of spike protein identical to Wuhan-1 in early December in Italy, among other evidence (van Dorp et al. 2020; Giovanetti et al. 2020; Xingguang Li et al. 2020; Amendola et al. 2021). The sequenced segment of the spike protein is short (409 bases), and it does not span positions in which 49 major early variants were observed, which means that the Italian Spike protein fragment can only confirm the existence of proCoV2 before the first coronavirus detection in China.

Our *tMRCA* is one month older than the date for the MRCA of genomes presented by Pekar et al. (2021) because their analysis is restricted to the ancestry of the coronaviruses sampled from China only that resulted in the exclusion of the ν lineage from their analysis. The potential for not sampling such lineages is well appreciated in Pekar et al. (2021). This exclusion and the use of sampling times in strict clock phylogenetic analyses would naturally lead to analyses leaning close to the earliest sampling times of SARS-CoV-2 (December 2019). Anyway, if we assume that proCoV2 was the index case, i.e., then the date of zoonosis (*tIndex*) would be late October to mid-November 2019. This range overlaps with Pekar et al.'s (2021) index date falling between mid-October and mid-November 2019. However, it likely that the actual *tIndex* is much earlier than *tMRCA* because proCoV2 likely increased in frequency over time before reaching a human host, and it is possible that one of its ancestors first infected a human. Based on an approximately one-month difference between *MRCA* and *Index* dates in Pekar et al. (2021), it is tempting to speculate that *tIndex* could have been as early as September 2019 for our SARS-CoV-2 phylogeny. This speculation requires more extensive analysis and confirmation in the future.

*Analysis of the 68KG database snapshot*

Next, we analyzed a later snapshot of SARS-CoV-2 genome collection acquired three months after the 29KG dataset. This dataset expanded the collection of coronavirus genomes from viral isolates collected after July 7, 2020 (16,739 genomes) and added 20,004 genome sequences from viral isolates dated before July 7, 2020. In the expanded MOA analysis, we retained 49 variants found with frequency >1% in the 29KG dataset and added variants found with a frequency >1% in the 68KG dataset (84 total variants; see Supplementary Table S1). MOA analysis of the 68KG dataset produced the proCoV2 genome identical to that inferred using the 29KG dataset (see *Materials and Methods*). We found one additional genome (EPI_ISL_493171) with a proCoV2 fingerprint sampled in Hubei, China, four weeks after the Wuhan-1 strain was reported.

The inferred mutation history from the 68KG dataset was well-supported with high COIs and BCLs and concordant with the mutation history produced using the 29KG dataset (**Fig. 4**). Therefore, inferences reported above for the 29KG dataset were robust to the expanded sampling of genomes. In the expanded mutation history, two new groups of variants were identified (**ζ** and **η**). They originated in mid-March 2020 and were found in a relatively high frequency in the 68KG dataset (~4.4% and 8.0%, respectively; Supplementary Table S1). Variants in **ζ** and **η** groups also showed episodic accumulation of mutations, e.g., the count of genomes containing three **ζ** mutations ($ζ_1$ - $ζ_3$; 2,955 genomes) was much larger than those with a subset of these variants (148 genomes). The episodic nature of mutational spread for 84 variants in the 68KG is statistically significant ($P < 10^{-8}$), i.e., clusters of mutations together have become common variants (see *Materials and Methods*).

## Coronavirus fingerprints and spatiotemporal tracking

The mutation history progression directly transforms into a collection of genetic fingerprints. Each fingerprint represents a genome type containing all the variants on the path from that tip node up to the progenitor proCoV2. These fingerprints can classify genomes and track spatiotemporal patterns of dominant lineages (see *Materials and Methods*). We use a shorthand to refer to each barcode in which only the major variant type is used. For example, **α** fingerprint refers to genomes that one or more of the α variants and no other major variants, and **αβ** fingerprint refers to genomes that contain at least one **α**, at least one **β** variant, and no other major variants. This nomenclature is intuitive and provides a way to glean evolutionary information from the coronavirus lineage's name. In the 68KG dataset (October 12, 2020 GISAID snapshot), global frequencies of major proCoV2 fingerprints were **αβε** (32.1%), **αβγδ** (17.7%), **αβ** (16.7%), **αβεη** (9.9%), **αβ** (9.8%), **αβγ** (6.8%), **αβζ** (4.5%), and **ν** (2.4%).

**Figure 5** shows the evolving spatiotemporal of all major fingerprints in Asia, Europe, and North America inferred for an expanded dataset of 172,480 genomes (December 30, 2020 snapshot). Spatiotemporal patterns in cities, countries, and other regions are available online at http://sars2evo.datamonkey.org/. We observe the spread and replacement of prevailing strains in Europe (**αβε** with **αβζ**) and Asia (**α** with **αβε**), the preponderance of the same strain for most of the pandemic in North America (**αβγδ**), and the continued presence of multiple high-frequency strains in Asia and North America. Spatiotemporal patterns of strain spread converged for Europe and Asia by July-August 2020 to **αβε** genetic fingerprints. These patterns diverged from North America, where **αβ** along with its mutant (**αβγδ**) were common. After that, Europe saw **ζ** variants of **αβ** grow (**αβζ**), replacing **αβε** genomes and its new **η** offshoot (**αβεη**)

(e.g., Hodcroft et al. 2020). The ζ mutations were first detected three weeks after the sampling of the first ε variants. Remarkably, αβγδ has remained the dominant lineage in North America since April 2020, in contrast to the turn-over seen in Europe and Asia.

More recently, novel fast-spreading variants have been reported (e.g., Rambaut et al. 2020). In particular, an S protein variant (N501Y) from South Africa and the UK has rapidly increased (Rambaut et al. 2020). Coronaviruses with N501Y variant in South Africa carry the αβγδ genetic fingerprint, whereas those in the UK carry the αβε genetic fingerprint. This means that the N501Y mutation arose independently in two coronavirus lineages that show convergent patterns of increased spread. At present, αβζ dominates the UK, and the number of genomes publicly available from South Africa is relatively small to make reliable inferences at present (see http://sars2evo.datamonkey.org for future updates). Overall, our mutational fingerprinting and nomenclature provide a simple way to glean the ancestry of new variants as compared to phylogenetic designations, e.g., B.1.351 and B.1.1.7 (Rambaut et al. 2020).

## Conclusions

Through innovative analyses of two large collections of SARS-CoV-2 genomes, we have consistently reconstructed the same progenitor coronavirus genome and identified its presence worldwide for many months after the pandemic began. The progenitor genome is a better reference for rooting phylogenies, orienting mutations, and estimating sequence divergences. The reconstructed mutational history of SARS-CoV-2 revealed major mutational fingerprints to identify and track the novel coronavirus's spatiotemporal evolution, revealing convergences and divergences of dominant strains among geographical regions from an analysis of more than 174 thousand genomes.

Furthermore, the approach taken here to reconstruct the progenitor genome and discover key mutational events will generally be applicable for analyzing pathogens during the early stages of outbreaks. The approach is scalable for even bigger datasets because it does not require more phylogenetically informative variants with an increasing number of samples. In fact, it benefits from bigger datasets as they afford more accurate estimates of individual and co-occurrence frequencies of variants and enable more reliable detection of lower frequency variants. Its continued application to SARS-CoV-2 genomes and other pathogen outbreaks will produce their ancestral genomes and their spatiotemporal dynamics, improving our understanding of the past, current, and future evolution of pathogens and associated diseases.

## Materials and Methods

Genome data acquisition and processing

A flowchart describing the protocol for data assembly and processing is shown in **Supplementary Figure S1**. In the first step, we download 60,332 SARS-CoV-2 genomes from the GISAID database (Shu and Mccauley 2017), along with information on sample collection dates and locations (until July 7, 2020). Of all the genomes downloaded, we only retained those with greater than 28,000 bases and were marked as originating from human hosts and passing controls detailed below. Similarly, the second dataset, the 68KG dataset, was assembled from 133,741 genomes and downloaded on October 12, 2020. Again, we retained only those with greater than 28,000 bases and marked as originating from human hosts.

Each genome was subjected to codon-aware alignment with the NCBI reference genome (accession number NC_045512) and then subdivided into ten regions based on CDS features: ORF1a (including nsp10), ORF1b (starting with nsp12), S, ORF3a, E, M, ORF6, ORF7a, ORF8, N, and ORF10. Gene ORF7b was removed because it was too short for alignment and comparisons. For each region, we scanned and discarded sequences containing too many ambiguous nucleotides to remove data with too many sequencing errors. Thresholds were 0.5% for the S gene, 0.1% for ORF1a and ORF1b genes, and 1% for all other genes. We mapped individual sequences to the NCBI reference genome (NC_045512) using a codon-aware extension to the Smith-Waterman algorithm implemented in HyPhy (Pond et al. 2005; Gianella et al. 2011) (https://github.com/veg/hyphy-analyses/tree/master/codon-msa), translated mapped sequence to amino-acids, and performed multiple protein sequence alignment with the auto settings function of MAFFT (version 7.453) (Katoh and Standley 2013). Codon sequences were next mapped onto the amino-acid alignment. The multiple sequence alignment of SARS-CoV-2 genomes was aligned with the sequence of three closest outgroups, including the coronavirus genomes of the *Rhinolophus affinis* bat (RaTG13), *R. malayanus* bat (RmYN02), and *Manis javanica* pangolin (MT121216.1) (Liu et al. 2020; Zhou et al. 2020). The alignment was visually inspected and adjusted in Geneious Prime 2020.2.2 (https://www.geneious.com). The final alignment contained all genomic regions except ORF7b and non-coding regions (5' and 3' UTRs, and intergenic spacers). After these filtering and alignment steps, the multiple sequence alignment contained 29,115 sites and 29,681 SARS-CoV-2 genomes for the July 7, 2020 snapshot, which we refer to as the 29KG dataset. For the October 12 snapshot, there were 68,057 sequences, which we refer to as the 68KG dataset. We also conducted a spatiotemporal analysis on an expanded dataset containing 172,480 genomes (172KG) acquired on December 30, 2020.

Reference genomes and collection dates

We used the dates of viral collections provided by the GISAID database (Shu and Mccauley 2017) in all our analyses if they were resolved to the day (i.e., we discarded data that only contained partial dates, e.g., April 2020). All genomes were used in the mutation ordering analyses, but genomes with incomplete sampling dates were excluded from the spatiotemporal analyses and derived interpretations. We noted that the earliest sample included in GISAID (ID: EPI_ISL_402123) was collected on December 24, 2019, although the NCBI website lists its collection date as December 23, 2019 (GenBank ID: MT019529). Therefore, we used the GISAID collection date for the sake of consistency. Regarding the NCBI reference genome (GenBank ID: NC_045512; GISAID ID: EPI_ISL_402125) (Wu et al. 2020), this sample was collected on December 26, 2019 (Chiara et al. 2021). We also used the GISAID reference genome in our analysis (ID: EPI_ISL_402124) collected on December 30, 2019 (Okada et al. 2020).

Mutation order analyses (MOA)

First, we analyzed the 29KG dataset. We used a maximum likelihood method, SCITE (Jahn et al. 2016), and variant co-occurrence analyses for reconstructing the order of mutations corresponding to 49 common variants (frequency > 1%) observed in this dataset (see flowchart in **Supplementary Figure S2**). MOA has demonstrated high accuracy for analyzing tumor cell genomes that reproduce clonally, have frequent sequencing errors, and exhibit limited sequence divergence (Jahn et al. 2016; Miura et al. 2018). In MOA, higher frequency variants are expected to have arisen earlier than low-frequency variants in clonally reproducing populations (Kim and Simon 2014; Jahn et al. 2016). We used the highest frequency variants to anchor the analysis and the shared co-occurrence of variants among genomes to order mutations while allowing probabilistically for sequencing errors and pooled sequencing of genomes (Jahn et al. 2016). MOA is different from traditional phylogenetic approaches where positions are treated independently, i.e., the shared co-occurrence of variants is not directly utilized in the inference procedure. Notably, both traditional phylogenetic and mutation order analyses are expected to produce concordant patterns when sequencing errors and other artifacts are minimized. However, sequencing errors and limited mutational input during the coronavirus history adversely impact traditional methods, as does the fact that the closest coronaviruses useable as outgroups have more than a thousand base differences from SARS-CoV-2 genomes that only differ in a handful of bases from each other (Mavian et al. 2020; Morel et al. 2020; Pipes et al. 2020).

MOA requires a binary matrix of presence/absence (1/0) of mutants, which is straightforward in analyzing cell sequences from tumors because they arise from normal cells that supply the definitive ancestral state. To designate mutation orientations for applying MOA in SARS-CoV-2 analysis, we devised a simple approach in which we began by comparing nucleotides at the 49 genomic positions among three closely related genomes (bat RaTG13, bat RmYN02, and pangolin MT121216.1) (Boni et al. 2020). We chose the consensus base to be the initial reference base, such that SARS-CoV-2 genome bases were coded to be "0" whenever they were the same as the consensus base at their respective positions. All other bases were assigned a "1." There were 39 positions in which all three outgroup genomes were identical to each other and 9 in which two of the outgroups showed the same base. In the remaining position (28657), all three outgroups differed, so we selected the base found in the gene with the highest sequence similarity to the human SARS-CoV-2 NCBI reference genome (NC_045512)

because SARS-CoV-2's ancestor likely experienced genomic recombination before its zoonotic transfer into humans (Huang et al. 2020; Xiaojun Li et al. 2020; MacLean et al. 2021). At one position, both major and minor bases in humans were different from the consensus base in the outgroups, so we assigned the mutant status to the minority base (U; *vf* = 29.8%). All missing and ambiguous bases were coded to be ignored (missing data) in all the analyses. These initially assigned mutation orientations were tested in a subsequent investigation of variants' co-occurrence index (COI). COI for a given variant ($y$) is the number of genomes that contain $y$ and its directly preceding mutation ($x$) in the mutation history, divided by the number of genomes that contain $y$. When COI was lower than 70%, we reversed each position's mutation orientation individually and selected the mutation orientation that produced mutation histories with the highest COI (see below).

In the SCITE analysis of 49 variants and 29,861 genomes, we started with default parameter settings of false-negative rate (FNR = 0.21545) and false-positive rate (FPR = 0.0000604) of mutation detection. We carried out five independent runs to ensure stability and convergence to obtain 29KG collection-specific estimates of FNR and FPR by comparing the observed and predicted sequences based on this mutation graph. The estimated FNR (0.00488) and FPR (0.00800) were very different from the SCITE default parameters, where the estimated FNR was much lower. This difference in error rates is unsurprising because we used only common variants (*vf* > 1%), and the 29KG dataset was not obtained from single-cell sequencing in which dropout during single-cell tumor sequencing elevates FNR, i.e., mutant alleles are not sequenced.

As noted above, the initial mutation orientations were simply the starting designations for our analysis, which are subsequently investigated by evaluating the COI of each variant in the reconstructed mutation history. In this process, we reverse ancestor/mutant coding for variants that received low COI to examine if a mutation history with higher COI can be generated. Two positions (3037 and 28854) received low COI (<70%). At position 3037, the reversed encoding (C→U) received significantly higher COI (100%) than the starting encoding (U→C; 60%), so the position was recoded. At position 28854, the ordering and direction of mutation remained ambiguous despite extensive analyses, but it did not impact the predicted MRCA sequence. Therefore, we only recoded the column for position 3037 and generated a new 49 × 29861 (SNVs × genomes) matrix to conduct a SCITE analysis.

At one position (28657), all three outgroup sequences had different bases, so we initially selected the base found in the gene with the highest sequence similarity to the human SARS-CoV-2 NCBI reference genome. We next tested if reversed encoding produced a better mutation graph. The reversed encoding produced a mutation graph with a much higher log-likelihood, i.e., −32355.58 and −30289.92, for the initial and reversed encoding, respectively; $P << 0.01$ using the AIC protocol in Pupko et al. (2002). Therefore, we recoded position 28657 and generated a new 49 × 29861 (SNVs × genomes) matrix.

It was subjected to SCITE analysis and produced a mutation graph for 49 variants in the 29KG dataset. This graph predicts an FNR of 0.00418 and FPR of 0.00295 per base. Using these new FNR and FPR, we again performed SCITE analysis and produced the final mutation history graph. Starting from the top of a mutation graph, a distinct Greek symbol was assigned to a group of mutations that were occurred sequentially, and variants with similar frequency were assigned the same Greek symbol (μ, ν, α, β, γ, δ, and ε). The high-frequency variants with the same Greek symbol were distinguished by numbers to represent the sequential relationship, e.g., $\alpha_1$ and $\alpha_2$. When an offshoot of a high-frequency mutation had low variant frequency, we assigned it the same Greek symbol and number to represent the parent-offspring relationship and further distinguished descendants by adding a small letter, e.g., $\alpha_{1a}$ and $\alpha_{1b}$.

Timing of the progenitor genome

The most recent common ancestor (MRCA) corresponds to the progenitor that gave rise to ν and α lineages in the mutation graph. MRCA is the progenitor of all human SARS-CoV-2 infections (proCoV2), which descended from the parental lineage that divergence form and its closest relatives, including bats and pangolins. We estimate that proCoV2 existed 5.8 to 8.1 weeks before December 24, 2019, on which the Wuhan-1 was sampled, by using a SARS-CoV-2 HPD mutation rate range of $6.64\times10^{-4}$ – $9.27\times10^{-4}$ substitutions per site per year (Pekar et al. 2021).

We have made available the proCoV2 genome sequence in FastA format at http://igem.temple.edu/COVID-19, which is the same as the NCBI reference genome with base differences corresponding to $\alpha_1 - \alpha_3$ mutations at positions 18060, 8782, and 28144, as discussed in the main text. In this mutation graph, COI for each variant is shown next to the arrow.

Bootstrap analysis

We assessed the robustness of the mutation history inference to genome sampling by bootstrap analysis. We generated 100 bootstrap replicate datasets, each built by randomly selecting 29,861 genomes with replacement. Then, SCITE was used to infer the mutation graph for each replicate dataset. Bootstrap confidence level, scored for each variant pair, was the number of replicates in which the given pair of variants were directly connected in the mutation history in the same way as shown in figure 2. BCLs were often lower for major variants within groups (e.g., $\varepsilon_1 - \varepsilon_3$) because they occur with very similar frequencies. This feature adversely affected the BCL values of mutation orders between groups, e.g., β and ε. In this case, we considered each group as a single entity. We computed BCL to be the proportion of replicates in which pairs of groups were directly connected in the mutation history in the same way as shown in figure 2. Groups used were $\beta_1$ - $\beta_3$, $\varepsilon_1$ - $\varepsilon_3$, and $\alpha_{1a}$ - $\alpha_{1d}$. All of these BCL values are shown with an underline.

Temporal concordance

Because MOA analyses did not use spatial or temporal information for genomes or mutations, the inferred mutation history can be validated by evaluating the concordance of the inferred order of mutations with the timing of their first appearance (*tf*). Using the genomes for which virus sampling day, month, and year were available, we determined *tf* for every variant in the 29KG dataset. For mutation *i*, we compared its *tf*(*i*) with *tf*(*j*) such that *j* is the nearest preceding mutation in the mutation graph. We found that $tf(j) \geq tf(i)$ for 47 of 49 mutations, except for $\beta_{3b}$ and $\beta_{3c}$ pairs. These two offshoot mutants of $\beta_3$ were sampled 35 days ($\beta_{3b}$) and 12 days ($\beta_{3c}$) earlier than their predecessors, which could be due to their low frequency or sequencing error. COI of one variant ($\beta_{3b}$) was low (54%), but the other variant ($\beta_{3c}$) had a very high COI (97%).

Mutational fingerprints

Each node in the mutational history graph predicts an intermediate (ancestral) or a tip sequence containing all the mutations from that node to the mutation graph's root. The mutational fingerprint is then produced directly from the mutation history graph drawn as a directional graph anchored on the root node. We compared our mutational fingerprints of the genomes in the 29KG dataset with a phylogeny-based classification (Rambaut et al. 2020) obtained using the Pangolin service (v2.0.3; https://pangolin.cog-uk.io/). We assigned each of the 29K genomes to a fingerprint based on the highest sequence similarity at positions containing 49 common variants, allowing mismatches due to population-level variations and sequencing errors. A small fraction of genomes (1.8%) could not be assigned unambiguously to one fingerprint, so they were excluded and investigated in the future. The number of genomes assigned to each fingerprint is shown in Table 1. We submitted genome sequences to the Pangolin website for classification one-by-one, and a clade designation was received. The results are summarized in Supplementary Figure S3. In this table, all phylogenetic groups with fewer than 20 genomes were excluded.

Of the 80 phylogenetic groups shown, 74 are defined primarily by a single mutation-based fingerprint, as more than 90% of the genomes in those phylogenetic groups share the same fingerprint. This includes all small and medium-sized phylogenetic groups (up to 488 genomes) and two large groups (A.1 with 1,377 genomes and B.1.2 with 749 genomes). One large group, B.1.1, predominately connects with $\varepsilon_3$ node (79%, 4,832 genomes), but some of its members belong to $\varepsilon_3$ offshoots because they contain respective diagnostic mutations. For group B.1.1.1, two other $\varepsilon_3$ offshoots are mixed up almost equally. Three other large differences between mutational fingerprint-based classification and phylogeny-based grouping are seen for A, B, B1.1, and B.2 groups. These differences are likely because the location of the root and the earliest branching order of coronavirus lineages are problematic in phylogeny-based classifications (Mavian et al. 2020; Morel et al. 2020; Pipes et al. 2020; Wenzel 2020). Overall, our mutational fingerprints are immediately informative about the mutational ancestry of genomes.

Analysis of 68KG dataset

We repeated the above MOA procedure on the 68KG dataset (68,057 genomes). This 68KG data contained 72 common variants (>1% frequency). For direct comparison purposes, we added 12 variants that were common variants on 29KG data, but their frequency had become less than 1% in the 68KG data. Therefore, we used 84 variants in total and constructed a matrix of 84 × 68,057 (SNVs × genomes) for the SCITE analysis to determine the mutational order. We also conducted the bootstrap analysis and assigned mutational fingerprints using the procedure mentioned above. The number of genomes mapped to each fingerprint is listed in Supplementary Table S1.

Sequence classification for 172KG dataset

We developed a sequence classification protocol that first calls variants in a viral genome using proCoV2 as the reference sequence using a browser-based sequence alignment (bioseq npm package) based on the codebase of minimap2 (Li 2018). Then, it assigns the sequence to a path in the mutation graph with the highest concordance (Jaccard index). It is implemented in a simple browser-based tool, which shows the example output for ENA accession number MT675945 (Supplementary Figure S4; http://sars2evo.datamonkey.org, last access March 18, 2021). The classification is conducted on the client-side such that the researcher's data never leaves their personal computer.

Testing episodic spread of variants

We performed non-parametric Wald–Wolfowitz runs-tests (Wald and Wolfowitz 1940; Mateus and Caeiro 2015) of the null hypothesis that the first sampling of variants is randomly distributed over time (i.e., evenly spaced). The null hypothesis was rejected for both 29KG and 64KG analysis at $P << 0.01$, suggesting significant temporally clustering in both 29KG dataset and 64KG datasets. Because many mutations were first sampled on December 24, 2019, we only included one mutation for that day to avoid biasing the test.

We also tested the null hypothesis of the same molecular evolutionary patterns within the SARS-CoV-2 population and between species (i.e., Human SARS-CoV-2 and Bat RaTG13) by using a McDonald-Kreitman test (McDonald and Kreitman 1991). The numbers of nonsynonymous and synonymous polymorphisms with a frequency greater than 1% were 32 and 17, compared with the numbers of nonsynonymous and synonymous fixed differences (170 and 958, respectively) inferred between proCoV2 and bat RaTG13 sequences. The McDonald-Kreitman test rejected the null overwhelmingly in a 2×2 contingency table analysis ($P << 0.01$).

## Data Availability and Code Availability

Live evolutionary history and spatiotemporal distributions of common variants can be accessed via http://sars2evo.datamonkey.org/. All genome sequences and metadata are available publicly at GISAID (https://www.gisaid.org/), and the predicted proCoV2 sequence is available at http://igem.temple.edu/COVID-19. The other relevant information is provided in the supplementary materials.

## Acknowledgments

We thank all the authors and organizations who have kindly deposited and shared genome data on GISAID (see http://igem.temple.edu/COVID-19 for a list of all the authors). We thank Ananias Escalante, Rob Kulathinal, Li Liu, Jose Barba-Montoya, Antonia Chroni, Ravi Patel, and Caryn Babaian for their critical comments. We appreciate the technical support provided by Jared Knoblauch and Glen Stecher. This research was supported by grants from the U.S. National Science Foundation to S.K. (GCR-1934848, DEB-2034228) and S.P. (DBI-2027196) and from the U.S. National Institutes of Health to S.K. (GM-139504-01) and S.P. (AI-134384).

## References

Amendola A, Bianchi S, Gori M, Colzani D, Canuti M, Borghi E, Raviglione MC, Zuccotti GV, Tanzi E. 2021. Evidence of SARS-CoV-2 RNA in an Oropharyngeal Swab Specimen, Milan, Italy, Early December 2019. *Emerg Infect Dis* 27.

Andersen KG, Rambaut A, Lipkin WI, Holmes EC, Garry RF. 2020. The proximal origin of SARS-CoV-2. *Nat Med* 26:450–452.

Boni MF, Lemey P, Jiang X, Lam TTY, Perry BW, Castoe TA, Rambaut A, Robertson DL. 2020. Evolutionary origins of the SARS-CoV-2 sarbecovirus lineage responsible for the COVID-19 pandemic. *Nat Microbiol* 5:1408–1417.

Casals F, Bertranpetit J. 2012. Human genetic variation, shared and private. *Science* 336:39–40.

Castells M, Lopez-Tort F, Colina R, Cristina J. 2020. Evidence of increasing diversification of emerging SARS-CoV-2 strains. *J Med Virol* 92:2165–2172.

Chiara M, Horner DS, Gissi C, Pesole G. 2021. Comparative genomics reveals early emergence and biased spatio- temporal distribution of SARS-CoV-2. *Mol Biol Evol*:msab049.

Dearlove BL, Lewitus E, Bai H, Li Y, Reeves DB, Joyce MG, Scott P, Amare M, Vasan S, Michael NL, et al. 2020. A SARS-CoV-2 vaccine candidate would likely match all currently circulating strains. *bioRxiv*, doi:10.1101/2020.04.27.064774.

van Dorp L, Acman M, Richard D, Shaw LP, Ford CE, Ormond L, Owen CJ, Pang J, Tan CCS, Boshier FAT, et al. 2020. Emergence of genomic diversity and recurrent mutations in SARS-CoV-2. *Infect Genet Evol* 83:104351.

Fauver JR, Petrone ME, Hodcroft EB, Shioda K, Ehrlich HY, Watts AG, Vogels CBF, Brito AF, Alpert T, Muyombwe A, et al. 2020. Coast-to-coast spread of SARS-CoV-2 during the early epidemic in the United States. *Cell* 181:990-996.e5.

Forster P, Forster L, Renfrew C, Forster M. 2020. Phylogenetic network analysis of SARS-CoV-2 genomes. *Proc Natl Acad Sci U S A* 117:9241–9243.

Gianella S, Delport W, Pacold ME, Young JA, Choi JY, Little SJ, Richman DD, Kosakovsky Pond SL, Smith DM. 2011. Detection of minority resistance during early HIV-1 infection: natural variation and spurious detection rather than transmission and evolution of multiple viral variants. *J Virol* 85:8359–8367.

Giorgio S Di, Martignano F, Torcia MG, Mattiuz G, Conticello SG. 2020. Evidence for host-dependent RNA editing in the transcriptome of SARS-CoV-2. *Sci Adv* 6:1–9.

Giovanetti M, Benvenuto D, Angeletti S, Ciccozzi M. 2020. The first two cases of 2019-nCoV in Italy: Where they come from? *J Med Virol* 92:518–521.

Gómez-Carballa A, Bello X, Pardo-Seco J, Martinón-Torres F, Salas A. 2020. Mapping genome variation of SARS-CoV-2 worldwide highlights the impact of COVID-19 super-spreaders. *Genome Res* 30:1434–1448.

Hodcroft EB, Zuber M, Nadeau S, Comas I, González Candelas F, Stadler T, Neher RA. 2020. Emergence and spread of a SARS-CoV-2 variant through Europe in the summer of 2020. *medRxiv*. doi:10.1101/2020.10.25.20219063.

Huang J-M, Jan SS, Wei X, Wan Y, Ouyang S. 2020. Evidence of the Recombinant Origin and Ongoing Mutations in Severe Acute Respiratory Syndrome Coronavirus 2 (SARS-CoV-2). *bioRxiv*. doi:10.1101/2020.03.16.993816.

Jackson B, Rambaut A, Pybus OG, Robertson DL, Connor T, Loman NJ. 2020. Recombinant SARS-CoV-2 genomes involving lineage B.1.1.7 in the UK. Available from: https://virological.org/t/recombinant-sars-cov-2-genomes-involving-lineage-b-1-1-7-in-the-uk/658 (last access March 24, 2021).

Jahn K, Kuipers J, Beerenwinkel N. 2016. Tree inference for single-cell data. *Genome Biol* 17:1–17.

Katoh K, Standley DM. 2013. MAFFT multiple sequence alignment software version 7: Improvements in performance and usability. *Mol Biol Evol* 30:772–780.

Kim KI, Simon R. 2014. Using single cell sequencing data to model the evolutionary history of a tumor. *BMC Bioinformatics* 15:27.

Komissarov AB, Safina KR, Garushyants SK, Fadeev A V., Sergeeva M V., Ivanova AA, Danilenko DM, Lioznov D, Shneider O V., Shvyrev N, et al. 2021. Genomic epidemiology of the early stages of the SARS-CoV-2 outbreak in Russia. *Nat Commun* 12:1–13.

Lai A, Bergna A, Acciarri C, Galli M, Zehender G. 2020. Early phylogenetic estimate of the effective reproduction number of SARS-CoV-2. *J Med Virol* 92:675–679.
Lemey P, Hong SL, Hill V, Baele G, Poletto C, Colizza V, O'Toole Á, McCrone JT, Andersen KG, Worobey M, et al. 2020. Accommodating individual travel history and unsampled diversity in Bayesian phylogeographic inference of SARS-CoV-2. *Nat Commun* 11:1–14.
Lemieux JE, Siddle KJ, Shaw BM, Loreth C, Schaffner SF, Gladden-Young A, Adams G, Fink T, Tomkins-Tinch CH, Krasilnikova LA, et al. 2021. Phylogenetic analysis of SARS-CoV-2 in Boston highlights the impact of superspreading events. *Science* 371:eabe3261.
Li H. 2018. Minimap2: pairwise alignment for nucleotide sequences. *Bioinformatics* 34:3094–3100.
Li Xiaojun, Giorg EE, Marichannegowda MH, Foley B, Xiao C, Kong XP, Chen Y, Gnanakaran S, Korber B, Gao F. 2020. Emergence of SARS-CoV-2 through recombination and strong purifying selection. *Sci Adv* 6:1–12.
Li Xingguang, Wang W, Zhao X, Zai J, Zhao Q, Li Y, Chaillon A. 2020. Transmission dynamics and evolutionary history of 2019-nCoV. *J Med Virol* 92:501–511.
Liu P, Jiang JZ, Wan XF, Hua Y, Li L, Zhou J, Wang X, Hou F, Chen Jing, Zou J, et al. 2020. Are pangolins the intermediate host of the 2019 novel coronavirus (SARS-CoV-2)? *PLoS Pathog* 16:1–13.
Lu R, Zhao X, Li J, Niu P, Yang B, Wu H, Wang W, Song H, Huang B, Zhu N, et al. 2020. Genomic characterisation and epidemiology of 2019 novel coronavirus: implications for virus origins and receptor binding. *Lancet* 395:565–574.
MacLean OA, Lytras S, Weaver S, Singer JB, Boni MF, Lemey P, Kosakovsky Pond SL, Robertson DL. 2021. Natural selection in the evolution of SARS-CoV-2 in bats, not humans, created a highly capable human pathogen. *PLoS Biol* 19:e3001115.
De Maio N, Walke C, Borges R, Weilguny L, Slodkowicz G, Goldman N. 2020. Issues with SARS-CoV-2 sequencing data. Available from: https://virological.org/t/issues-with-sars-cov-2-sequencing-data/473 (last access March 24, 2021).
Martin D, Weaver S, Tegally H, San J, Wilkinson E, Giandhari J, Pillay Y, Singh L, Lessells RJ, Oliveira T De, et al. 2021. The emergence and ongoing convergent evolution of the N501Y lineages coincided with a major global shift in the SARS-CoV-2 selective landscape. *medRxiv*, doi:10.1101/2021.02.23.21252268.
Mateus A, Caeiro F. 2015. An R implementation of several randomness tests.Simos ZK, Monovasilis T, editors. *AIP Conf Proc* 1618:531–534.
Mavian C, Pond SK, Marini S, Magalis BR, Vandamme AM, Dellicour S, Scarpino S V., Houldcroft C, Villabona-Arenas J, Paisie TK, et al. 2020. Sampling bias and incorrect rooting make phylogenetic network tracing of SARS-COV-2 infections unreliable. *Proc Natl Acad Sci U S A* 117:12522–12523.
McDonald JH, Kreitman M. 1991. Adaptive protein evolution at the Adh locus in Drosophila. *Nature* 351:652–654.
Miura S, Huuki LA, Buturla T, Vu T, Gomez K, Kumar S. 2018. Computational enhancement of single-cell sequences for inferring tumor evolution. *Bioinformatics* 34:i917–i926.
Morel B, Barbera P, Czech L, Bettisworth B, Huebner L, Lutteropp S, Serdari D, Kostaki E-G, Mamais I, Kozlov A, et al. 2020. Phylogenetic analysis of SARS-CoV-2 data is difficult. *Mol Biol Evol*:msaa314.
Nei M, Kumar S. 2002. Molecular Evolution and Phylogenetics. Oxford University Press
Okada P, Buathong R, Phuygun S, Thanadachakul T, Parnmen S, Wongboot W, Waicharoen S, Wacharapluesadee S, Uttayamakul S, Vachiraphan A, et al. 2020. Early transmission patterns of coronavirus disease 2019 (COVID-19) in travellers from Wuhan to Thailand, January 2020. *Euro Surveill* 25:2000097.
Pekar J, Worobey M, Moshiri N, Scheffler K, Wertheim JO. 2021. Timing the SARS-CoV-2 index case in Hubei province. *Science*. doi:10.1126/science.abf8003.
Pipes L, Wang H, Huelsenbeck J, Nielsen R. 2020. Assessing uncertainty in the rooting of the SARS-CoV-2 phylogeny. *Mol Biol Evol*:msaa316.
Pond SLK, Frost SDW, Muse S V. 2005. HyPhy: hypothesis testing using phylogenies. *Bioinformatics* 21:676–679.
Pupko T, Huchon D, Cao Y, Okada N, Hasegawa M. 2002. Combining multiple data sets in a likelihood analysis: Which models are the best? *Mol Biol Evol* 19:2294–2307.
Rambaut A, Holmes EC, O'Toole Á, Hill V, McCrone JT, Ruis C, du Plessis L, Pybus OG. 2020. A dynamic nomenclature proposal for SARS-CoV-2 lineages to assist genomic epidemiology. *Nat Microbiol* 5:1403–1407.
Rambaut A, Loman N, Pybus O, Barclay W, Barrett J, Carabelli A, Connor T, Peacock T, Robertson DL, Volz E. 2020. Preliminary genomic characterisation of an emergent SARS-CoV-2 lineage in the UK

defined by a novel set of spike mutations. Available from: https://virological.org/t/preliminary-genomic-characterisation-of-an-emergent-sars-cov-2-lineage-in-the-uk-defined-by-a-novel-set-of-spike-mutations/563 (last access March 24, 2021).

Rice AM, Morales AC, Ho AT, Mordstein C, Mühlhausen S, Watson S, Cano L, Young B, Kudla G, Hurst LD. 2020. Evidence for strong mutation bias towards, and selection against, U content in SARS-CoV-2: implications for vaccine design. *Mol Biol Evol* 38:67–83.

Richard D, Owen CJ, van Dorp L, Balloux F. 2020. No detectable signal for ongoing genetic recombination in SARS-CoV-2. *bioRxiv*. doi:10.1101/2020.12.15.422866.

Ross EM, Markowetz F. 2016. OncoNEM: Inferring tumor evolution from single-cell sequencing data. *Genome Biol* 17:1–14.

Shu Y, Mccauley J. 2017. GISAID: Global initiative on sharing all influenza data-from vision to reality. *Euro Surveill* 22:30494.

da Silva Filipe A, Shepherd JG, Williams T, Hughes J, Aranday-Cortes E, Asamaphan P, Ashraf S, Balcazar C, Brunker K, Campbell A, et al. 2021. Genomic epidemiology reveals multiple introductions of SARS-CoV-2 from mainland Europe into Scotland. *Nat Microbiol* 6:112–122.

Stefanelli P, Faggioni G, Lo Presti A, Fiore S, Marchi A, Benedetti E, Fabiani C, Anselmo A, Ciammaruconi A, Fortunato A, et al. 2020. Whole genome and phylogenetic analysis of two SARSCoV-2 strains isolated in Italy in January and February 2020: Additional clues on multiple introductions and further circulation in Europe. *Euro Surveill* 25:1–5.

Tang X, Wu C, Li X, Song Y, Yao X, Wu X, Duan Y, Zhang H, Wang Y, Qian Z, et al. 2020. On the origin and continuing evolution of SARS-CoV-2. *Natl Sci Rev* 7:1012–1023.

Tegally H, Wilkinson E, Giovanetti M, Iranzadeh A, Fonseca V, Giandhari J, Doolabh D, Pillay S, San EJ, Msomi N, et al. 2021. Emergence of a SARS-CoV-2 variant of concern with mutations in spike glycoprotein. *Nature*. doi:10.1038/s41586-021-03402–03409.

Turakhia Y, De Maio N, Thornlow B, Gozashti L, Lanfear R, Walker CR, Hinrichs AS, Fernandes JD, Borges R, Slodkowicz G, et al. 2020. Stability of SARS-CoV-2 phylogenies. *PLOS Genet* 16:e1009175.

Wald A, Wolfowitz J. 1940. On at test whether two samples are from the same population. *Ann Math Stat* 11:147–162.

Wenzel J. 2020. Origins of SARS-CoV-1 and SARS-CoV-2 are often poorly explored in leading publications. *Cladistics* 36:374–379.

Worobey M, Pekar J, Larsen BB, Nelson MI, Hill V, Joy JB, Rambaut A, Suchard MA, Wertheim JO, Lemey P. 2020. The emergence of SARS-CoV-2 in Europe and the US. *bioRxiv*. doi:10.1101/2020.05.21.109322.

Wu F, Zhao S, Yu B, Chen YM, Wang W, Song ZG, Hu Y, Tao ZW, Tian JH, Pei YY, et al. 2020. A new coronavirus associated with human respiratory disease in China. *Nature* 579:265–269.

Yang Z, Kumar S, Nei M. 1995. A new method of inference of ancestral nucleotide and amino acid sequences. *Genetics* 141:1641–1650.

Zhou P, Yang X Lou, Wang XG, Hu B, Zhang L, Zhang W, Si HR, Zhu Y, Li B, Huang CL, et al. 2020. A pneumonia outbreak associated with a new coronavirus of probable bat origin. *Nature* 579:270–273.

Table 1. SARS-CoV-2 variants in 29KG dataset.

| Mutant (major) | Mutant (minor) | Gene | Genomic Position | Nucleotide change | Amino acid change | Time (days) | Variant Frequency | Genomes mapped | First location |
|---|---|---|---|---|---|---|---|---|---|
| $\mu_1$ | | ORF1ab | 2416 | U>C | | 0 | 98.1% | 0 | China, Asia |
| $\mu_2$ | | ORF1ab | 19524 | U>C | | 0 | 98.6% | 0 | China, Asia |
| $\mu_3$ | | S | 23929 | U>C | | 0 | 98.4% | 18 | China, Asia |
| $\alpha_1$ | | ORF1ab | 18060 | U>C | | 0 | 95.1% | 849 | China, Asia |
| | $\alpha_{1a}$ | N | 28657 | C>U | | 63 | 1.3% | 2 | France, Europe |
| | $\alpha_{1b}$ | ORF1ab | 9477 | U>A | F>Y | 63 | 1.2% | 3 | France, Europe |
| | $\alpha_{1c}$ | N | 28863 | C>U | S>L | 63 | 1.2% | 5 | France, Europe |
| | $\alpha_{1d}$ | ORF3a | 25979 | G>U | G>V | 63 | 1.2% | 344 | France, Europe |
| $\alpha_2$ | | ORF1ab | 8782 | U>C | | 0 | 91.0% | 47 | China, Asia |
| $\alpha_3$ | | ORF8 | 28144 | C>U | S>L | 0 | 90.8% | 1115 | China, Asia |
| | $\alpha_{3a}$ | ORF1ab | 1606 | U>C | | 43 | 1.7% | 501 | United Kingdom, Europe |
| | $\alpha_{3b}$ | ORF1ab | 11083 | G>U | L>F | 24 | 9.2% | 376 | China, Asia |
| | $\alpha_{3c}$ | N | 28311 | C>U | P>L | 64 | 1.9% | 3 | South Korea, Asia |
| | $\alpha_{3d}$ | ORF1ab | 13730 | C>U | A>V | 71 | 1.8% | 3 | Taiwan/Malaysia, Asia |
| | $\alpha_{3e}$ | ORF1ab | 6312 | C>A | T>K | 71 | 1.7% | 483 | Taiwan/Malaysia, Asia |
| | $\alpha_{3f}$ | ORF3a | 26144 | G>U | G>V | 28 | 5.1% | 121 | China, Asia |
| | $\alpha_{3g}$ | ORF1ab | 14805 | C>U | | 54 | 6.0% | 334 | United Kingdom, Europe |
| | $\alpha_{3h}$ | ORF1ab | 17247 | U>C | | 64 | 2.0% | 580 | Switzerland, Europe |
| | $\alpha_{3i}$ | ORF1ab | 2558 | C>U | P>S | 54 | 1.7% | 26 | United Kingdom, Europe |
| | $\alpha_{3j}$ | ORF1ab | 2480 | A>G | I>V | 54 | 1.6% | 462 | United Kingdom, Europe |
| $\beta_1$ | | ORF1ab | 3037 | C>U | | 31 | 77.0% | 11 | China, Asia |
| $\beta_2$ | | S | 23403 | A>G | D>G | 31 | 77.1% | 36 | China, Asia |
| $\beta_3$ | | ORF1ab | 14408 | C>U | P>L | 41 | 76.9% | 3032 | Saudi Arabia, Middle East |
| | $\beta_{3a}$ | ORF1ab | 20268 | A>G | | 64 | 5.7% | 1213 | Italy, Europe |
| | $\beta_{3b}$ | N | 28854 | C>U | S>L | 29 | 3.1% | 527 | China, Asia |
| | $\beta_{3c}$ | ORF1ab | 15324 | C>U | | 29 | 2.3% | 678 | China, Asia |
| | $\beta_{3d}$ | ORF3a | 25429 | G>U | V>L | 77 | 1.7% | 485 | United Kingdom, Europe |
| | $\beta_{3e}$ | N | 28836 | C>U | S>L | 74 | 1.6% | 3 | Switzerland, Europe |
| | $\beta_{3f}$ | ORF1ab | 13862 | C>U | T>I | 74 | 1.6% | 50 | Switzerland, Europe |
| | $\beta_{3g}$ | ORF1ab | 10798 | C>A | D>E | 86 | 1.4% | 414 | United Kingdom, Europe |
| $\gamma_1$ | | ORF3a | 25563 | G>U | Q>H | 41 | 29.8% | 884 | Saudi Arabia, Middle East |
| | $\gamma_{1a}$ | ORF1ab | 18877 | C>U | | 41 | 4.0% | 757 | Saudi Arabia, Middle East |
| | $\gamma_{1b}$ | M | 26735 | C>U | | 41 | 1.5% | 439 | Saudi Arabia, Middle East |
| $\delta_1$ | | ORF1ab | 1059 | C>U | T>I | 54 | 23.0% | 5157 | Singapore, Asia |
| | $\delta_{1a}$ | S | 24368 | G>U | D>Y | 75 | 1.3% | 389 | Sweden, Europe |
| | $\delta_{1b}$ | ORF8 | 27964 | C>U | S>L | 76 | 2.7% | 790 | USA, North America |
| | $\delta_{1c}$ | ORF1ab | 11916 | C>U | S>L | 72 | 1.6% | 166 | USA, North America |
| | $\delta_{1d}$ | ORF1ab | 18998 | C>U | A>V | 72 | 1.0% | 305 | USA, North America |
| $\varepsilon_1$ | | N | 28881 | G>A | R>K | 54 | 25.7% | 2 | United Kingdom, Europe |
| $\varepsilon_2$ | | N | 28882 | G>A | R>K | 54 | 25.7% | 2 | United Kingdom, Europe |
| $\varepsilon_3$ | | N | 28883 | G>C | G>R | 54 | 25.7% | 5365 | United Kingdom, Europe |
| | $\varepsilon_{3a}$ | ORF1ab | 313 | C>U | | 66 | 2.1% | 608 | USA, North America |
| | $\varepsilon_{3b}$ | ORF1ab | 19839 | U>C | | 64 | 1.5% | 452 | Switzerland, Europe |
| | $\varepsilon_{3c}$ | M | 27046 | C>U | T>M | 69 | 1.6% | 453 | Worldwide |
| | $\varepsilon_{3d}$ | ORF1ab | 10097 | G>A | G>S | 69 | 2.5% | 5 | Denmark, Europe |
| | $\varepsilon_{3e}$ | S | 23731 | C>U | | 69 | 2.5% | 403 | Denmark, Europe |
| | $\varepsilon_{3f}$ | N | 28580 | G>U | D>Y | 69 | 1.2% | 353 | Chile, South America |
| $\nu_1$ | | ORF1ab | 17858 | A>G | Y>C | 59 | 4.7% | 32 | USA, North America |
| $\nu_2$ | | ORF1ab | 17747 | C>U | P>L | 59 | 4.7% | 1374 | USA, North America |

Note.- Genomic locations correspond to those of the NCBI genome (GenBank ID: NC_04551.2). Amino acid changes are shown for nonsynonymous variants.

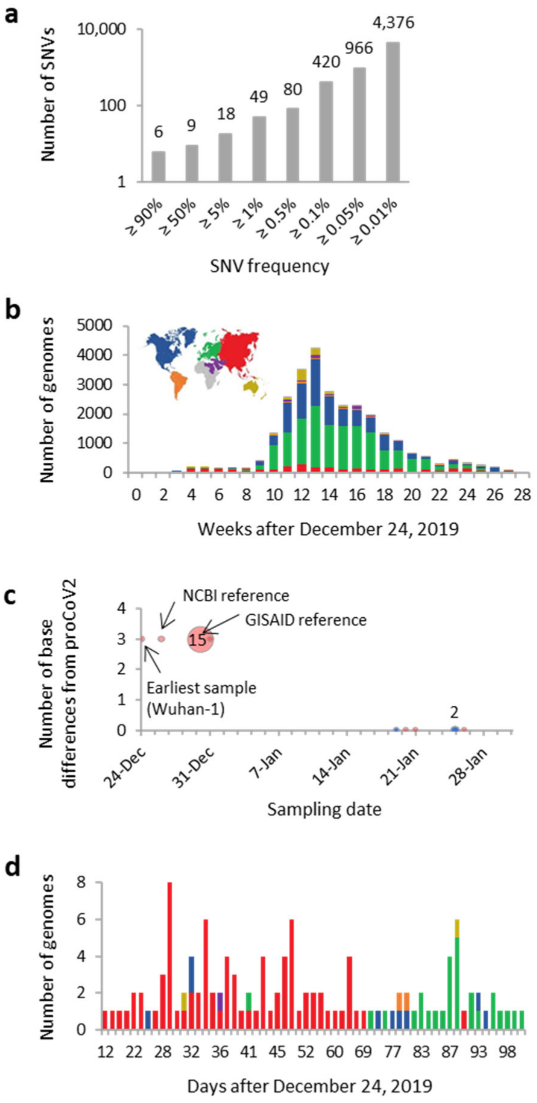

Figure 1. Counts of single nucleotide variants (SNVS) and genomes in the 29KG dataset. (a) Cumulative count of SNVs presented in the 29KG genome dataset at different frequencies. (b) The number of genomes in the 29KG collection that were isolated weekly during the pandemic. (c) The number of base differences from proCoV2 (see figure 2) for genomes sampled in December 2019 and January 2020. The 18 genomes sampled in December 2019 in China (red) have three common SNVs different from proCoV2. In contrast, six genomes sampled in January 2020 in China (Asia, red) and the USA (North America, blue) show no base differences. Multiple genomes (2 and 15) were sampled on two different days. (d) Temporal and spatial distribution of strains identical to proCoV2 at the protein sequence level, i.e., they have only μ mutations. The color scheme used to mark sampling locations is shown in panel b.

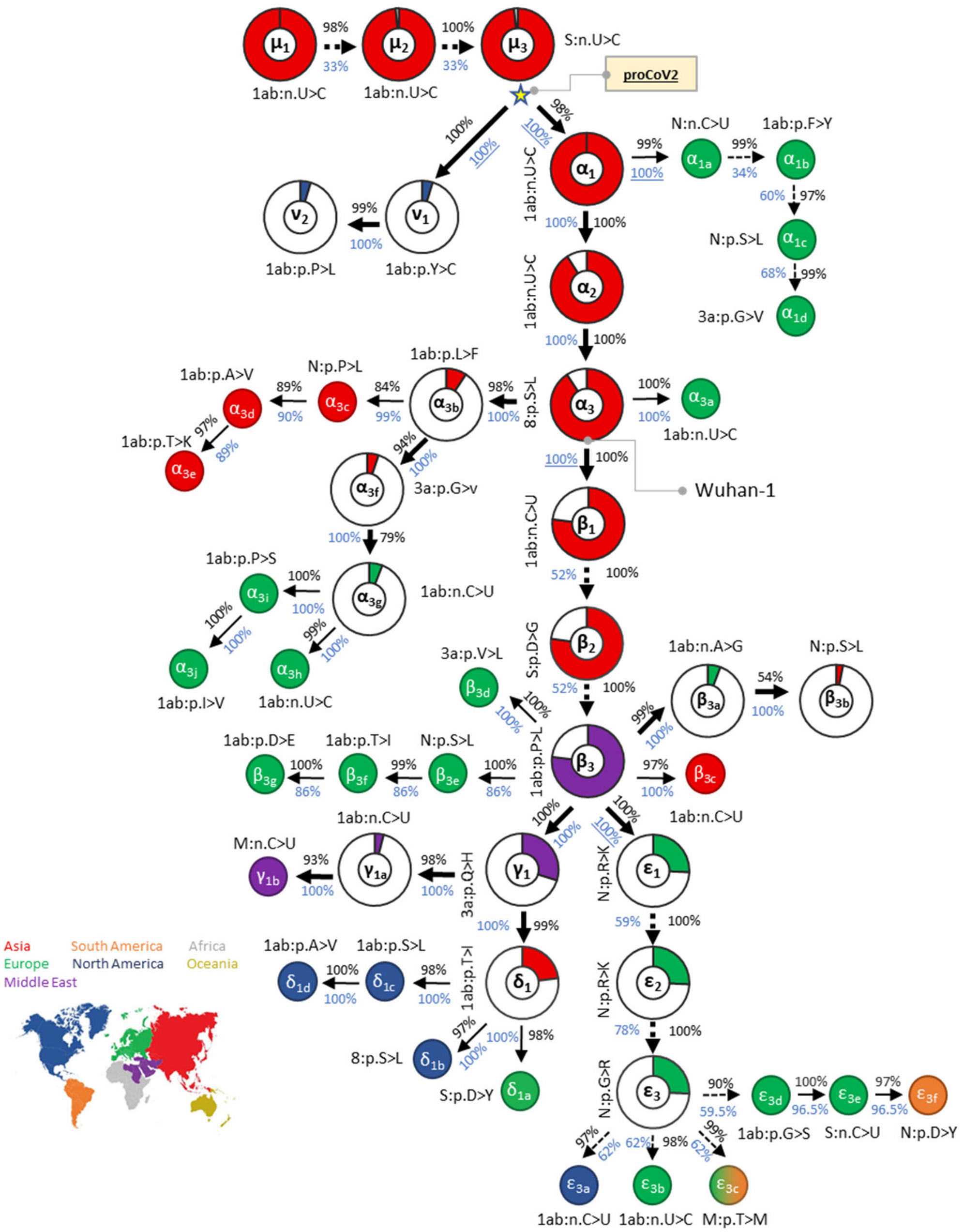

Figure 2. Mutational history graph of SARS-CoV-2 from the 29KG dataset. Thick arrows mark the pathway of widespread variants (frequency, *vf* ≥ 3%), and thin arrows show paths leading to other common mutations (3% > *vf* > 1%). The pie-chart sizes are proportional to variant frequencies in the 29KG dataset, with pie-charts shown for variants with *vf* > 3% and pie color based on the world's region where that mutation was first observed. A circle is used for all other variants, with the filled color corresponding to the earliest sampling region. The co-occurrence index (COI, black font) and the bootstrap confidence level (BCL, blue font) of each mutation and its predecessor mutation are shown next to the arrow connecting them. Underlined BCL values mark variant pairs for which BCLs were estimated for groups of variants (see Materials and Methods) because of the episodic nature of variant accumulation within groups resulting in lower BCLs (<80%; dashed arrows). Base changes (n.) are shown for synonymous mutations, and amino acid changes (p.) are shown for nonsynonymous mutations along with the gene/protein names ("ORF" is omitted from gene name abbreviations given in Table 1). More details on each mutation are presented in Table 1.

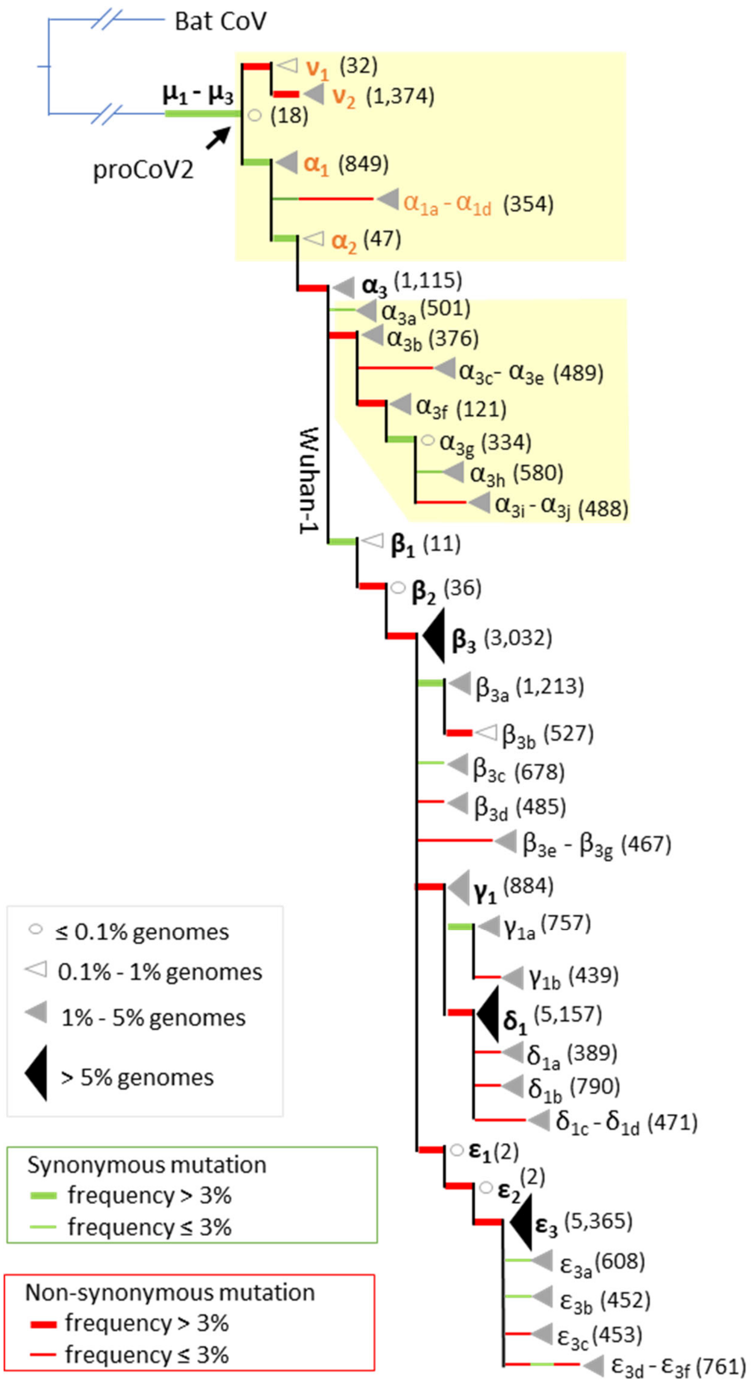

Figure 3. A waterfall display of genome phylogeny recapitulating the mutation history in figure 2. The numbers of genomes mapped to each node are depicted by open circles (very few genomes), open triangles (few genomes), small gray triangles (many genomes), and large black triangles (very many genomes). The actual number of genomes is given in the parenthesis. The tip label is the name of the mutation on the connecting branch. Green and red branches are synonymous and nonsynonymous mutations, respectively. Thick branches mark mutations that occur with a frequency greater than 3% in the 29KG data. The yellow background highlights the diversity of coronavirus lineages that evolved from the genomes leading to Wuhan-1 coronavirus.

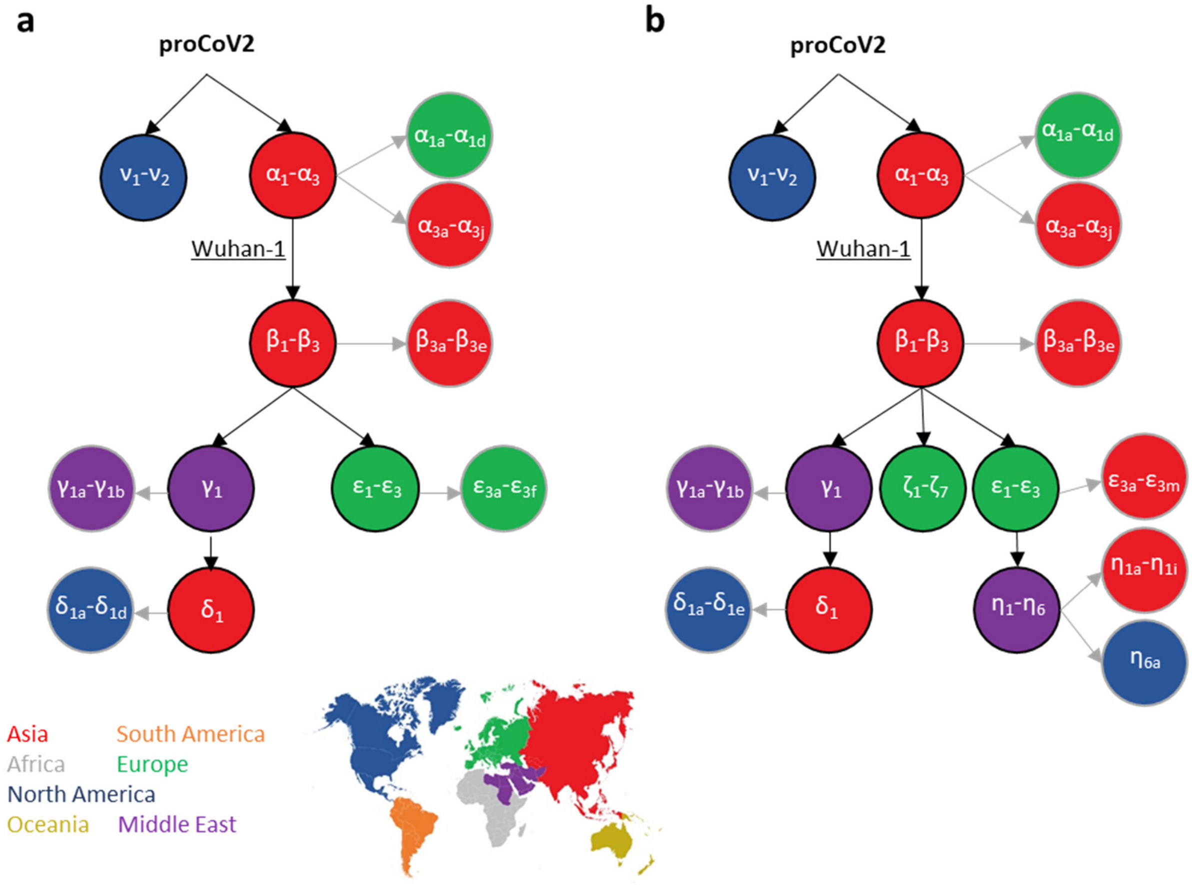

Figure 4. The backbone of SARS-CoV-2 mutational history. The mutational history inferred was from (a) 29KG and (b) 68KG datasets. Major variants and their mutational pathways are shown in black, and minor variants and their mutational pathways are gray. Circle color marks the region where variants were sampled first. The 68KG dataset contains 12 additional variants and more than two times the genomes than the 29KG dataset.

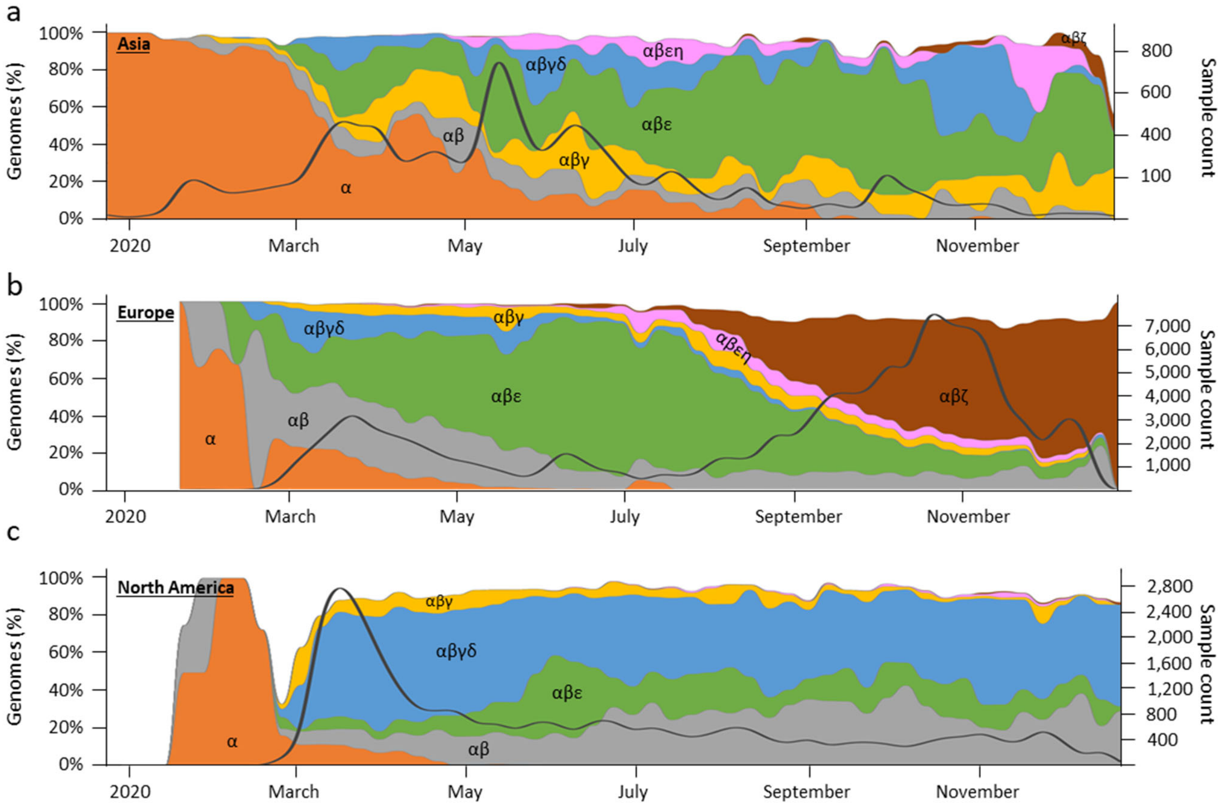

Figure 5. Spatiotemporal dynamics of 172,480 SARS-CoV-2 genomes (December 2019-2020). Spatiotemporal patterns of genomes mapped to lineages containing different combinations of major variants in (a) Asia, (b) Europe, and (c) North America. The number of genomes mapped to major variant lineages contains all of its offshoots, e.g., α lineage contains all the genomes with $\alpha_1 - \alpha_3$, $\alpha_{1a} - \alpha_{1d}$, and $\alpha_{3a} - \alpha_{3j}$ variants only. The stacked graph area is the proportion of genomes mapped to the corresponding lineage. The solid black line shows the count of total genome samples. Spatiotemporal patterns in cities, countries, and other regions are available online at http://sars2evo.datamonkey.org/ (last accessed on March 28, 2021).

Figure 5
