# Supplementary material for: An Evolutionary Portrait of the Progenitor SARS-CoV-2 and Its Dominant Offshoots in COVID-19 Pandemic
Source: Mol Biol Evol. 2021 May 4;38(8):3046–59. doi: 10.1093/molbev/msab118 (PMC8135569; doi:10.1093/molbev/msab118)
Supplement: msab118_Supplementary_Data [file msab118_supplementary_data.pdf]

Supplementary Table 1. SARS-CoV-2 variants in the 68KG dataset.

Mutant (major)	Mutant (minor)	Gene	Genomic Position	Nucleotide change	Amino acid change	Time (days)	Variant Frequency	Genomes mapped	First location
μ_1		ORF1ab	2416	U>C		0	98.4%	0	China, Asia
μ_2		ORF1ab	19524	U>C		0	99.0%	18	China, Asia
μ_3		S	23929	U>C		0	98.9%	0	China, Asia
μ_4		ORF1ab	15933	U>C		0	98.8%	0	China, Asia
μ_5		ORF8	27944	U>C		0	97.0%	0	China, Asia
μ_6		ORF1ab	6286	U>C		0	95.6%	0	China, Asia
μ_7		S	22444	U>C		0	98.7%	0	China, Asia
α_1		ORF1ab	18060	U>C		0	97.3%	1114	China, Asia
	α_{1a}	N	28657	C>U		63	1.0%	3	France, Europe
	α_{1b}	ORF1ab	9477	U>A	F>Y	63	0.7%	3	France, Europe
	α_{1c}	N	28863	C>U	S>L	63	0.7%	7	France, Europe
	α_{1d}	ORF3a	25979	G>U	G>V	63	0.7%	451	France, Europe
α_2		ORF1ab	8782	U>C		0	94.9%	51	China, Asia
α_3		ORF8	28144	C>U	S>L	0	94.9%	1281	China, Asia
	α_{3a}	ORF1ab	1606	U>C		43	0.9%	578	United Kingdom, Europe
	α_{3b}	ORF1ab	11083	G>U	L>F	24	7.5%	417	China, Asia
	α_{3c}	N	28311	C>U	P>L	64	1.4%	4	South Korea, Asia
	α_{3d}	ORF1ab	13730	C>U	A>V	33	1.4%	5	China, Asia
	α_{3e}	ORF1ab	6312	C>A	T>K	71	1.2%	767	Taiwan, Asia
	α_{3f}	ORF3a	26144	G>U	G>V	28	3.0%	160	China, Asia
	α_{3g}	ORF1ab	14805	C>U		54	3.7%	511	United Kingdom, Europe
	α_{3h}	ORF1ab	17247	U>C		64	1.0%	682	Switzerland, Europe
	α_{3i}	ORF1ab	2558	C>U	P>S	54	1.0%	44	United Kingdom, Europe
	α_{3j}	ORF1ab	2480	A>G	I>V	54	1.0%	648	United Kingdom, Europe
β_1		ORF1ab	3037	C>U		31	87.2%	45	China, Asia
β_2		S	23403	A>G	D>G	31	87.2%	15	China, Asia
β_3		ORF1ab	14408	C>U	P>L	41	87.1%	4450	Saudi Arabia, Middle East
	β_{3a}	ORF1ab	20268	A>G		64	6.0%	2388	Italy, Europe
	β_{3b}	N	28854	C>U	S>L	29	4.5%	1782	China, Asia
	β_{3c}	ORF1ab	15324	C>U		29	2.2%	1463	China, Asia
	β_{3d}	ORF3a	25429	G>U	V>L	77	1.1%	719	United Kingdom, Europe
	β_{3e}	N	28836	C>U	S>L	74	0.8%	3	Switzerland, Europe
	β_{3f}	ORF1ab	13862	C>U	T>I	74	0.8%	85	Switzerland, Europe
	β_{3g}	ORF1ab	10798	C>A		86	0.6%	435	United Kingdom, Europe
γ_1		ORF3a	25563	G>U	Q>H	41	24.4%	1671	Saudi Arabia, Middle East
	γ_{1a}	ORF1ab	18877	C>U		41	4.2%	1201	Saudi Arabia, Middle East
	γ_{1b}	M	26735	C>U		41	2.7%	1784	Saudi Arabia, Middle East
δ_1		ORF1ab	1059	C>U	T>I	54	17.6%	8284	Singapore, Asia
	δ_{1a}	S	24368	G>U	D>Y	75	0.7%	466	Sweden, Europe
	δ_{1b}	ORF8	27964	C>U	S>L	76	2.9%	1152	USA, North America
	δ_{1c}	ORF1ab	11916	C>U	S>L	72	1.9%	807	USA, North America
	δ_{1d}	ORF1ab	18998	C>U	A>V	72	0.7%	458	USA, North America
	δ_{1e}	ORF1ab	10319	C>U	L>F	76	1.2%	799	USA, North America
ζ_1		ORF1ab	445	U>C		179	4.4%	18	Netherlands, Europe
ζ_2		M	26801	C>G		82	4.3%	7	Canada, North America
ζ_3		S	22227	C>U	A>V	84	4.5%	1	Spain, Europe
ζ_4		N	28932	C>U	A>V	96	4.4%	5	Portugal, Europe
ζ_5		ORF10	29645	G>U	V>L	78	4.4%	2	Denmark, Europe
ζ_6		ORF1ab	21255	G>C		80	4.4%	1557	USA, North America
ζ_7		S	21614	C>U	L>F	79	2.5%	1442	United Kingdom, Europe

Supplementary Table 1. SARS-CoV-2 variants in the 68KG dataset (continued).

Mutant (major)	Mutant (minor)	Gene	Genomic Position	Nucleotide change	Amino acid change	Time (days)	Variant Frequency	Genomes mapped
ε_1		N	28881	G>A	R>K	54	41.7%	5
ε_2		N	28882	G>A	R>K	54	41.6%	0
ε_3		N	28883	G>C	G>R	54	41.6%	13394
	ε_{3a}	ORF1ab	313	C>U		64	2.4%	1630
	ε_{3b}	ORF1ab	19839	U>C		64	2.9%	1227
	ε_{3c}	M	27046	C>U	T>M	69	0.8%	548
	ε_{3d}	ORF1ab	10097	G>A	G>S	69	3.2%	11
	ε_{3e}	S	23731	C>U		69	3.2%	425
	ε_{3f}	N	28580	G>U	D>Y	69	1.0%	678
	ε_{3g}	ORF1ab	13536	C>U		69	1.6%	23
	ε_{3h}	ORF1ab	4002	C>U	T>I	69	1.6%	1066
	ε_{3i}	ORF1ab	10265	G>A	G>S	63	1.4%	879
	ε_{3j}	S	21575	C>U	L>F	54	1.0%	248
	ε_{3k}	S	21637	C>U		111	1.3%	873
	ε_{3l}	ORF8	28169	A>G		103	1.3%	0
	ε_{3m}	ORF1ab	16968	G>U		114	1.0%	702
η_1		ORF1ab	1163	A>U	I>F	86	9.6%	339
	η_{1a}	ORF1ab	14202	G>U		159	1.1%	7
	η_{1b}	ORF1ab	19542	G>U	M>I	81	1.2%	23
	η_{1c}	S	22388	C>U		90	1.2%	21
	η_{1d}	N	29466	C>U	A>V	91	1.2%	4
	η_{1e}	ORF1ab	19718	C>U	T>I	73	1.5%	23
	η_{1f}	ORF3a	26060	C>U	T>I	92	1.2%	7
	η_{1g}	N	29227	G>U		55	1.2%	24
	η_{1h}	ORF1ab	3256	U>C		167	1.1%	0
	η_{1i}	ORF1ab	5622	C>U	P>L	67	1.2%	775
η_2		ORF1ab	18555	C>U		51	8.0%	25
η_3		ORF1ab	16647	G>U		84	8.0%	8
η_4		ORF1ab	7540	U>C		86	7.9%	0
η_5		S	23401	G>A		86	7.9%	1
η_6		S	22992	G>A	S>N	86	8.5%	4583
	η_{6a}	S	22480	C>U		66	1.3%	878
ν_1		ORF1ab	17858	A>G	Y>C	59	2.6%	61
ν_2		ORF1ab	17747	C>U	P>L	59	2.5%	1677

Note.- Genomic locations correspond to those of the NCBI genome (GenBank ID: NC_04551.2). Amino acid changes are shown for nonsynonymous variants.

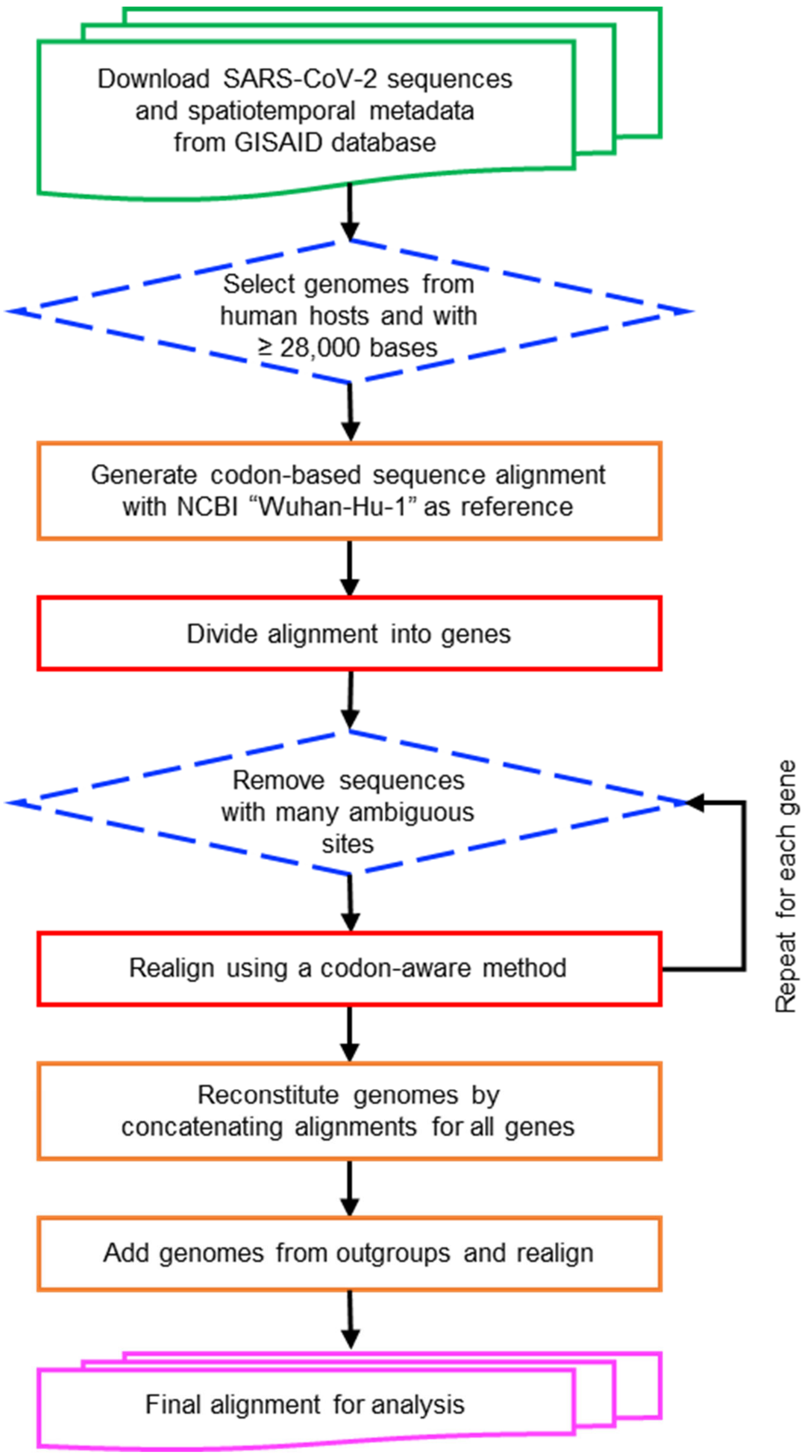

Supplementary Figure 1. Flowchart summarizing the procedure to generate two curated multiple sequence alignments used in this study (29KG and 68KG) from the SARS-CoV-2 genomes available in the GISAID database. Abbreviations are as follows: GISAID (Global Initiative on Sharing Avian Influenza Data [https://www.gisaid.org]); NCBI (National Center for Biotechnology Information [https://www.ncbi.nlm.nih.gov]).

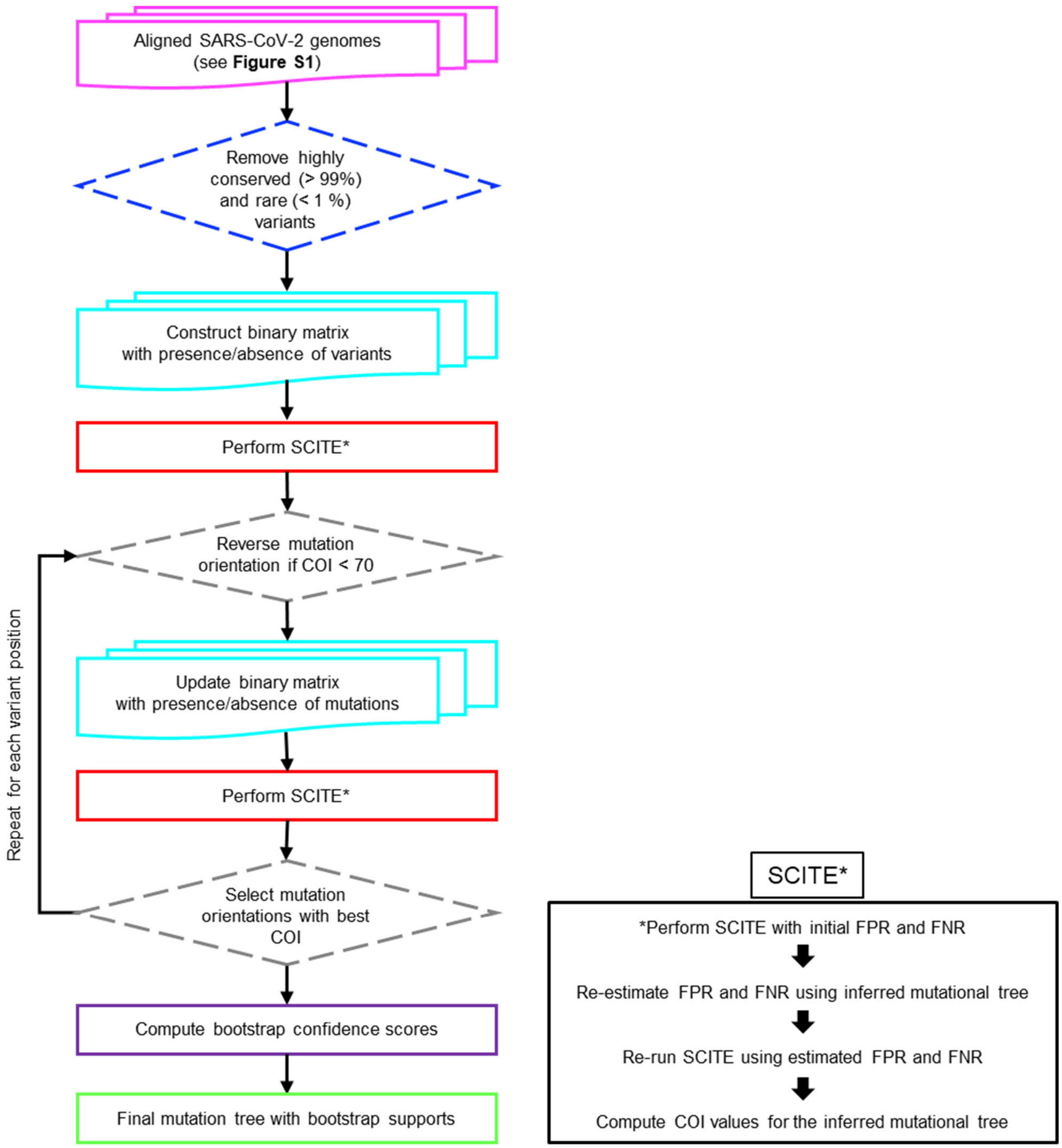

Supplementary Figure 2. Flowchart summarizing the steps performed to construct the mutation order tree from the curated SARS-CoV-2 alignments (i.e., 29KG and 68KG). Abbreviations are as follows: GISAID (Global Initiative on Sharing Avian Influenza Data); SCITE (Single Cell Inference of Tumor Evolution); FPR (False Positive Rates); FNR (False Negative Rates); COI (Co-Occurrence Index).

	Mutation-based fingerprints																																		
Phylo-class	α1	α1d	α2	α3	α3a	α3b	α3e	α3f	α3h	α3i	α3j	β1	β3	β3a	β3b	β3c	β3d	β3f	β3g	γ1	γ1a	δ1	δ1a	δ1b	δ1c	δ1d	ε3	ε3a	ε3b	ε3c	ε3e	ε3f	γ1b	ν1	ν2
B.1													2256	515	8	576	204	50		522	588	4410	369	41	165	268									
B.1.1																											4832	558	277	423					
A.1																																		15	1362
B				744	349	126						33		6	22	14																			
B.2				7				367	402																										
B.1.2																								749											
B.1.1.1																															403	342			
A	440		42			9																												16	
B.2.1										26	462																								
B.6							483																												
B.1.5													23	412																					
B.1.93																			414																
A.2		344																																	
B.3				275		11																													
B.1.36																																	252		
A.3	239																																		
B.1.11																	229																		
B.1.22																				205															
B.4				13		197																													
B.1.8													189																						
B.1.113																																	180		
B.1.43																						174													
B.1.1.33																											129								
B.1.1.10																											110								
B.1.26																						110													
B.2.2									107																										
A.5	87																																		
B.1.1.2																													90						
B.1.42																						90													
B.1.9																					90														
B.16					86																														
B.1.75													86																						
B.1.116																						80													
B.1.102														76																					
B.1.13																				67															
B.1.5.2														64																					
B.1.124																					62														
B.1.5.9													61																						
B.1.1.7																											53								
B.2.4								54																											
B.1.80																48																			
B.1.89													49																						
B.1.39																						48													
A.6	41																																		
B.1.107													43																						
B.1.110																				44															
B.1.78													44																						
B.1.5.5														43																					
B.1.1.4																													41						
B.2.7									40																										
B.1.79													39																						
B.1.1.28																											38								
B.1.3																										37									
B.1.37																				37															
B.1.5.10														37																					
B.1.104																	35																		
B.10					35																														
B.1.1.32																												31							
B.1.96													33																						
B.1.1.26																											32								
B.1.41																						32													
B.1.40																						30													
A.4	29																																		
B.1.1.30																											29								
B.1.19													29																						
B.1.1.25																											28								
B.1.12																						27													
B.14				27																															
B.1.1.14																											25								
B.1.1.5																													25						
B.1.101														25																					
B.1.108																						25													
B.1.117																						25													
B.1.23													25																						
B.1.1.17																														24					
B.2.6									22																										
B.1.120																						21													
B.1.29																						21													
B.1.71													21																						
B.1.44																							20												

Supplementary Figure 3. A comparison of mutation-based and phylogeny-based classifications of 29KG genomes. Phylogeny-based classification is obtained by using the Pangolin service (v2.0.3; https://pangolin.cog-uk.io/). Only the terminal variants are shown in mutation-based fingerprints for convenience. Each cell's value is the number of genomes that belong to the corresponding mutation-based and phylogeny-based groups. All phylogenetic-based groups with fewer than 20 genomes are excluded. Cells with fewer than five genomes matching have been left empty to make the comparison more straightforward and allow for sequencing and estimation errors.

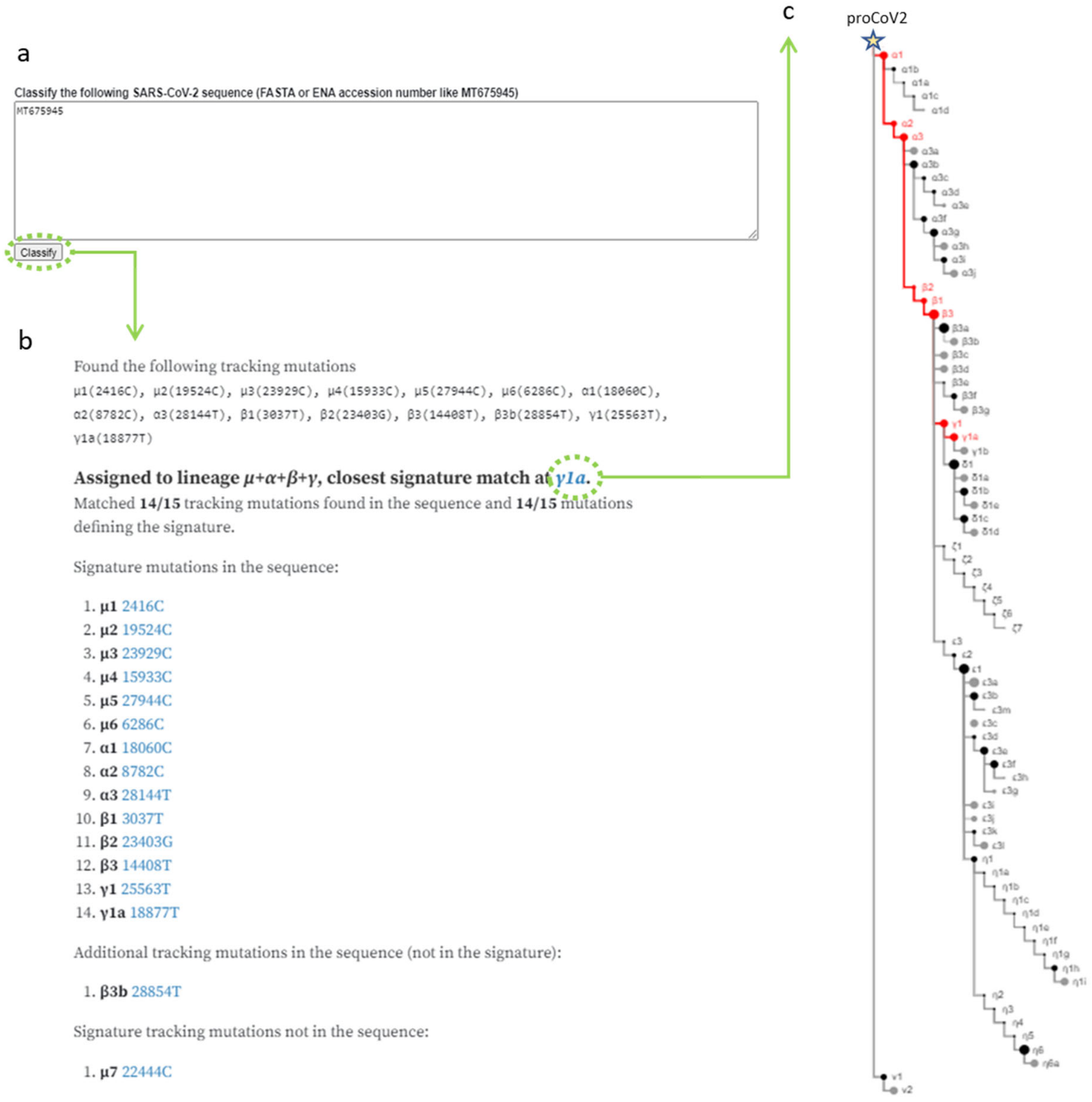

Supplementary Figure 4. An example of sequence classification (ENA Accession MT675945) based on the 84 signature mutations (http://sars2evo.datamonkey.org/; "Classify your Sequence" option). (a) Input window to provide identifiers of sequences to be classified (e.g., MT675945). (b) The input sequence is classified into a mutational fingerprint. A list of mutations that are appeared in the input sequence is shown in the output window. (c) A waterfall phylogeny shows the input sequence's location in the phylogeny, which appears after clicking the closet signature matched mutation in panel b.
